# Higher order structural effects stabilizing the reverse Watson–Crick Guanine-Cytosine base pair in functional RNAs

**DOI:** 10.1093/nar/gkt800

**Published:** 2013-10-08

**Authors:** Mohit Chawla, Safwat Abdel-Azeim, Romina Oliva, Luigi Cavallo

**Affiliations:** ^1^Physical Sciences and Engineering Division, Kaust Catalysis Center, King Abdullah University of Science and Technology, Thuwal 23955-6900, Saudi Arabia and ^2^Department of Sciences and Technologies, University of Naples ‘Parthenope’, Centro Direzionale Isola C4, I-80143, Naples, Italy

## Abstract

The G:C reverse Watson–Crick (W:W trans) base pair, also known as Levitt base pair in the context of tRNAs, is a structurally and functionally important base pair that contributes to tertiary interactions joining distant domains in functional RNA molecules and also participates in metabolite binding in riboswitches. We previously indicated that the isolated G:C W:W trans base pair is a rather unstable geometry, and that dicationic metal binding to the Guanine base or posttranscriptional modification of the Guanine can increase its stability. Herein, we extend our survey and report on other H-bonding interactions that can increase the stability of this base pair. To this aim, we performed a bioinformatics search of the PDB to locate all the occurencies of G:C trans base pairs. Interestingly, 66% of the G:C trans base pairs in the PDB are engaged in additional H-bonding interactions with other bases, the RNA backbone or structured water molecules. High level quantum mechanical calculations on a data set of representative crystal structures were performed to shed light on the structural stability and energetics of the various crystallographic motifs. This analysis was extended to the binding of the preQ1 metabolite to a preQ1-II riboswitch.

## INTRODUCTION

RNA molecules fold giving rise to complex structures that exhibit evolutionary conserved architectures. These rely on a variety of stabilizing strategies, with base stacking interactions and base–base, base–backbone and backbone–backbone H-bonding interactions as the most important stabilizing forces (1–6). As well-known, besides the canonical Watson–Crick base pairs, RNA bases can give several ‘noncanonical’ H-bonding interactions, by either using the Watson–Crick edge in a reversed way or involving their ‘Sugar’ or ‘Hoogsteen’ edges (7–9). These noncanonical interactions offer a structural flexibility to folding strategies, and it is maybe not by chance that they are often included in higher order structural motifs, such as H-bonded base triplets and quartets. Furthermore, other factors, such as structured water molecules, metal ions, posttranscriptional modifications or protonation of a nucleobase, can affect the geometry and stability of specific motifs in RNA molecules (10–20).

This variety of structural solutions spurred a large amount of experimental and theoretical studies devoted to characterize energetically and geometrically each of these interactions, with the general idea that they can be considered as building blocks contributing to the overall structure and stability of the nucleic acids. Among theoretical methods, quantum mechanics (QM)-based approaches revealed fundamental in the accurate characterization of small structural units, such as simple H-bonded base pairs, which are otherwise difficult to characterize experimentally. This approach has been possible because, in the large majority of the cases, the gas phase optimization of isolated pairs results in a geometry that is consistent with the experimental one, reflecting the intrinsic geometrical stability of the base pairing interaction and allowing to use these methods to quantify the energetics of the interaction (11,16,21–28). On this basis, when a large discrepancy occurs between the calculated geometry of a base pair and the geometry of the same base pair in experimental structures, this evidence has been taken as an indication that other effects contribute to the stability of the specific base pair geometry in the experimental structure.

In this context, we previously showed that the G:C W:W trans (or reverse Watson–Crick) base pair, known to mediate the interaction between the D-loop and the V-loop in tRNA structures, seems not to be intrinsically stable from a geometrical point of view (29). Instead of a G:C W:W trans, the gas phase optimized G15–C48 geometry is a bifurcated one, involving the central section of the G Watson–Crick face and the C carbonyl group adjacent to the C1’, which is classifiable as a G–C Ww/Bs trans [according to the Leontis and Westhof nomenclature as extended by Lemieux and Major for bifurcated geometries, (8,30)]. This bifurcated base pair is highly nonisosteric with the G:C W:W trans base pair, having a different orientation of the glycosidic bonds and a distance between the C1’ atoms of the two bases 1.6 Å shorter. We also found the occurrence of such a bifurcated geometry in experimental structures of RNA molecules (12). These results are consistent with experiments indicating a reduced stability of the W:W trans geometry for the G:C pair. For example, it has been shown that the introduction of G:C W:W trans base pairs in all A-T parallel strand DNA duplexes, characterized by W:W trans base pairs (31,32), significantly reduces the duplex thermodynamic stability, both for base stacking and hydrogen bonding contributions (33,34).

Nevertheless, despite of this geometrical instability, the G:C W:W trans base pair is found to be recurrent not only in transfer but also in 23S ribosomal RNA structures. To complete this scenario, we note that a G:C W:W trans pair has been recently shown to be involved in the recognition of the metabolite preQ_1_ by the class II preQ_1_ riboswitch (35). The recurrence of a geometrically unstable base pair is somewhat surprising. However, we previously suggested that possible additional factors could help the G:C W:W trans pair to be held in place (12,14). Focusing on this point, we have previously shown that the binding of a divalent metal ion to the N7 atom of the guanine, or the archaeosine (7-formamidino-7-deazaguanosine) posttranscriptional modification (36), acting as a ‘covalent mimic’ of the metal binding to the N7 atom, can stabilize the G:C W:W trans geometry in the context of the tRNA structure (12).

**Figure 1.**
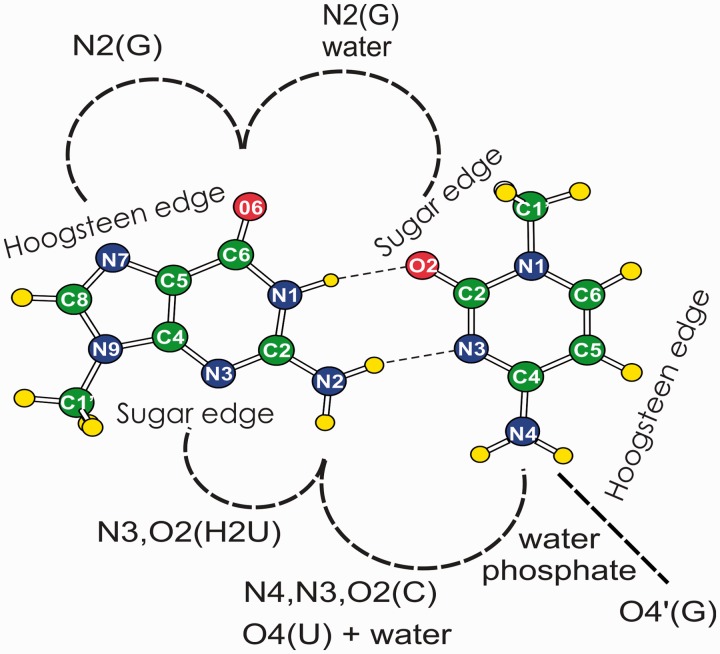
The G:C W:W trans pair with a schematic representation of clamping interactions provided by surrounding moieties.

Considering that our previous work indicated that the G:C W:W trans pair geometrical stability can be improved by metal binding or posttranscriptional modification, we decided to extend the analysis to search for other environmental factors that could act similarly. We started with a thorough search of the wwPDB (37), looking for occurrences of the G:C W:W trans pairs involved in H-bonding interactions with other bases, the ribose-phosphate backbone and water molecules. On the basis of this search, we show here that the G:C W:W trans pair possesses natural propensity to participate in more complex interaction patterns, like base triplets and quartets, taking advantage of H-bonds capability of its ‘Hoogsteen edges’ (through Guanine O6 and N7 and Cytosine N4, C5 and C6) and ‘Sugar edges’ (through Guanine O2’, N2 and N3 and Cytosine O2’ and O2) (see Figure 1). Then, a representative of each experimentally observed structural motif was subjected to high level QM calculations, to investigate its geometry and stability. The QM approach adopted here has been thoroughly validated in the study of the structure and energetics of H-bonded bases in several nucleic acid systems (11,20–22,38–44). Finally, using this knowledge, we decompose the binding of the preQ_1_ metabolite by the *Lactobacillus rhamnosus* class II preQ_1_ riboswitch (35).

Based on this approach, combining structural bioinformatics and accurate QM calculations, we address the following fundamental questions: (i) how many types of higher order structures (viz. triplet or quartets) involving G:C W:W trans base pair as a core motif are present in RNA 3D structures; (ii) how the formation of higher order structures influences the geometric stability of the G:C W:W trans pair; (iii) what are the other environmental factors that provide H-bonding interactions that may affect the geometric stability of G:C W:W trans pairs in RNA structures.

## MATERIALS AND METHODS

### Nomenclature used in the present work

Leontis and Westhof proposed a nomenclature for H-bonded base pairs based on the three interacting edges, i.e. Watson–Crick, Hoogsteen or sugar and the two mutual orientations of the glycosidic bonds, i.e cis or trans (7,8). Similarly, a more extended nomenclature has been recently proposed for triplet motifs (45). According to this nomenclature, after the one-letter nucleotide symbol, a small ‘c’ or ‘t’ is given, to indicate that the orientation of the glycosidic bonds is cis or trans, respectively, followed by a capital ‘W’, ‘H’ or ‘S’, to indicate that the ‘Watson–Crick’, ‘Hoogsteen’ or ‘Sugar’ edge is involved in the base-base H-bonding interaction. A small ‘r’ precedes the one-letter nucleotide symbol, in case the corresponding ribose is involved in H-bonding interactions. This is a standard nomenclature used in literature (11,18).

### Structural analysis of higher order motif interactions involving G:C trans as core structure

The subset of PDB structures used in this work, updated to September 2012, is composed of the 1487 crystallographic structures containing RNA molecules solved at a resolution of 3.5 Å or better. These structures were analyzed using the BPview tool (46), to look for the G:C W:W trans base pairing geometry and its associated higher order H-bonded array motifs. The analyzed ensemble was kept redundant because, as shown in the following, even structures of the same RNA molecule can present different geometries of the G:C trans pair. False positives were excluded from the analysis by visual inspection. Geometries of the G:C trans pair different from the W:W trans were also assigned by visual inspection. The complete list of higher order interaction motifs observed in PDB structures and involving a G:C base pair with a trans orientation as a core is provided in the Supplementary Materials (Supplementary Table S1). An in-house script was used to detect the interaction of the G:C trans base pair with water molecules and the phosphate backbone.

## COMPUTATIONAL DETAILS

### Model building

The initial model of each of the structural motifs subject to QM calculations was built from selected examples from the PDB data set. The PDB IDs and the corresponding nucleotide IDs used are given in Table 1. The ribose is not included, unless it participates in H-bonding interactions with the G:C bases, so that the models of the bases are normally truncated at the level of the C1’ atom of the ribose. In the cases where a ribose sugar is involved in H-bonding interactions, the ribose ring is included in the model and thus the nucleotides are terminated by replacing the –CH_2_-5’OH and the -3’OH groups with a methyl group, to mimic the phosphodiester linkage between consecutive nucleotides. This is the standard approach used in the literature (18,21,23,40).

**Table 1.** All computed interactions are reported with the corresponding PDB code and resolution, of the selected crystal structure

Motif	Occurrency	Interaction	PDB; Biomolecule	Resolution (Å)	Crystallographic G:C motif
GCC tWW/tSW	9	G15:C48:C20	1H3E; tRNA^Tyr^	2.90	W:W trans
GCU tWW/Intermediate-O4(U)	10	G:515:C548:U520	1N78; tRNA^Glu^	2.10	W:W trans
GCH2U(rG) tWW/tSW	1	G15:C48:H2U20A:G20B	1SER;tRNA^Ser^	2.90	W:W trans
GrCrG tWW/cSS	74	G430:C234:G219	3uz2; 23S rRNA	2.80	W:W trans
GGCU(i) tHS/tWW/tWW	17	G1371:G1360:C2214: U2210	1VS8; 23S rRNA	3.50	W:W trans
GGCU(ii) tHS/tWW/tWW	5	G1371:G1360:C2214: U2210	3OAS; 23S rRNA	3.25	Ww/Bs trans
GGCU(iii) tHS/tWW/tWW	8	G1371:G1360:C2214: U2210	2AW4; 23S rRNA	3.46	Ww/Bw trans
GC tWW/wc	160	G15:C48:w916	1EVV; tRNA^Phe^	2.00	W:W trans
GC tWW/wa	82	G1873:C1856:w9741	1YIJ; 23S rRNA	2.60	W:W trans
GC tWW/wcwa	68	G15:C48:w104:w121	1EHZ; tRNA^Phe^	1.93	W:W trans
GC tWW/OP1	269	G2564:C2510:PO1 2508	1FFK; 23S rRNA	2.40	W:W trans
GC tWW/OP1wc	34	G2564:C2510:PO1(C2508)w3705	3CCL; 23S rRNA	2.90	W:W trans

### QM calculations

A density functional theory approach, based on the hybrid B3LYP functional as implemented in the gaussian09 package (47–49) and the cc-pVTZ basis set (50), was used for all geometry optimizations, both in the gas phase and in water, modeled with the continuum polarizable model C-PCM (51,52). For the systems involving phosphate groups of the backbone interacting with base pairs, the cc-pVTZ basis set for the phosphate atoms was augmented by a diffuse polarization function. Interaction energies were calculated on the B3LYP/cc-pVTZ geometries at the second order Møller-Plesset, MP2, level of theory (53) using the cc-pVTZ basis sets (50). For these calculations, we used the RIMP2 method as implemented in the Turbomole 6.1 package, and the solvent was modeled with the COSMO continuum model (54). All interaction energies were corrected for Basis Set Superposition Error (BSSE), using the counterpoise procedure (55).

Because the aim of this work is to understand the stabilization of the G:C W:W trans geometry by interaction with other surrounding bases, water or sugar, we focus on the interaction energy between the G:C W:W trans base pair and the surrounding moieties. For reasons that will become clear in the following, we define this interaction energy as ‘clamping energy’, E_clamp_, calculated as in Equation (1):
(1)
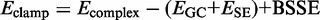

where *E*_complex_ is the electronic energy of the whole system, *E*_GC_ and *E*_SE_ are the electronic energies of the isolated geometry of the G:C W:W trans base pair and of the surrounding environment residues, respectively, frozen to the geometry they have in the optimized complex, and BSSE is the basis set superimposition error. This is a standard approach used in this kind of calculation (16,56–59). In addition, we calculated the interaction energy between the G and C base pairs in the geometry they have in each optimized complex. This interaction energy, *E*_GC_, is calculated as in Equation (2):
(2)
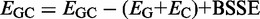

where, *E*_GC_ is the energy of the G:C base pair in the geometry it has in the optimized complex, while *E*_G_ and *E*_C_ are the energy of the isolated and optimized G and C bases, and BSSE is again the basis set superimposition error.

## RESULTS

### Identification of motifs involving G:C W:W trans base pairs in RNA 3D structures

Using the BPview tool, a search across the 1487 selected crystal structures containing RNA molecules was carried out to record all possible higher order motifs such as triplets and quartets that may possibly involve a G:C trans base pair, see Table 1. In total, we detected 1780 instances of G:C pairs classified as trans by the BPview program. However, after further geometrical checking, 667 entries were found to be false positives (i.e. they do not present the H-bond pattern of a W:W trans or a W:W Ww/Bs geometry). Of the remaining 1113 instances, 738 are involved in additional H-bonding interactions as those described below, while 375 represent simple G:C trans pairs with no additional H-bonding interaction. This simple analysis indicates that roughly two-third of the G:C trans entries in the wwPDB are engaged in additional H-bonding interactions, highlighting their relevance.

**Figure 2.**
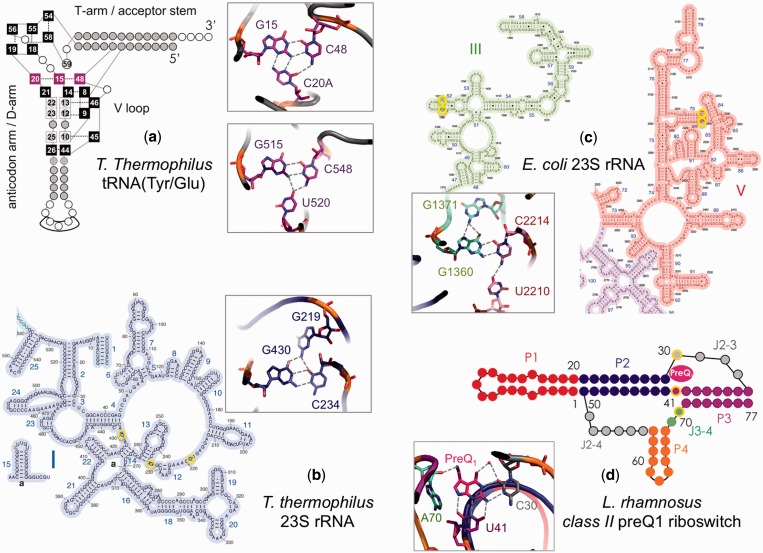
Examples of higher order motifs including the G:C W:W trans pair as a core, in the context of the secondary/tertiary structure of functional RNAs. (**a**) The GCC tWW/tSW and GCU tWW/Intermediate-O4(U) triplets, mediating the interaction between the D-loop and the V-loop at the elbow of *T.thermophilus* tRNAs(Tyr/Glu); (**b**) the GrCrG tWW/cSS triplet, connecting three different junctions of Domain I in *T.thermophilus* 23S rRNA; (**c**) the GGCU(i) tHS/tWW/tWW quartet, joining domains III and V in *E. coli* 23S rRNA; and (**d**) the binding site of the preQ1 metabolite, in a three-way junction between the P2, P3 and P4 stems of *L.rhamnosus* class II preQ1 riboswitch.

More specifically, our analysis indicated the occurrence of four distinct triplet motifs, where the third residue interacts with both the guanine and cytosine residues of the G:C trans pair (no instances were found where the third base is H-bonded to only one of the bases of the G:C pair), and three quartet motifs, where one of the additional bases interacts with the guanine, whereas the other one interacts with the cytosine. Further, we found a series of instances where the G:C pair is interacting with structured water molecules, the ribose or the phosphate of the RNA backbone, see Table 1. A schematic representation of the various motifs is shown in Figure 2, while the frequency of occurrence is reported in Table 1 together with the specification of the representative structure used in the QM calculations. The complete list of occurrences of the G:C trans base pairing geometries in the examined PDB structures is given in Supplementary Table S1.

The first triplet is a CGC tWW/tSW motif, where an additional cytosine, uses its ‘Watson–Crick’ edge to pair with the ‘Sugar’ edge of the guanine and the N4 atom of the cytosine in the G:C pair. We found nine instances of this motif, all in tRNA molecules. The second triplet is a CGU tWW/Intermediate-O4(U) motif, where the O4 atom of the uracil is involved in H-bonds with both the cytosine and the guanosine of the G:C pair, thus mediating directly the interaction between the G:C W:W trans pair. We recorded 10 instances of this motif, all in tRNA molecules. The third triplet is a CGH2U(rG) tWW/tSW motif, where the 5,6-dihydro-U residue uses its ‘Watson–Crick’ edge to pair with the ‘Sugar’ edge of the guanine. We could detect a single instance of this motif, in the crystal structure of the class II (i.e. long V-loop) tRNA^Ser^ from *Thermus thermophilus*. All the above three triplets are involved in mediating the interaction between the D-loop and the V-loop in the tRNA molecules (See Figure 2a).

The fourth triplet is a GrCrG tWW/cSS motif, where an additional guanine uses its ‘Sugar edge’ to pair with the ‘Sugar edge’ of the cytosine in the G:C W:W trans pair. We observed 74 instances for this triplet, 73 of them corresponding to the G430:C234:G219 motif in structures of the 23S rRNA from *T. thermophilus*. All the residues of this motif belong to domain I of 23S rRNA. Interestingly, G430 is located in junction 4/14, while C234 is in junction 12/13, and G219 in junction 11/12. Thus, such interaction joins together different junction loops, aiding the folding of the ribosome. The remaining instance of this triplet corresponds to the G458:C224:G209 motif, again in the 23 s rRNA from *T. thermophilus* structure (Figure 2b).

The quartet motifs that we observed are all of the type GGCU tHS/tWW/tWW. This motif is characterized by a core G:C W:W trans base pair, with the additional guanine H-bonding to the Hoogsten edge of the guanine in the trans pair, and the fourth base, a uracil, H-bonding to the amino group of the cytosine Watson–Crick edge. We recorded 30 instances of this motif, all in structures of 23 s rRNA from *Escherichia coli*, specifically as G1371:G1360:C2214:U2210. Interestingly, G1360 and G1371 are located in an internal loop of helix 52 in the 23 S Domain III, whereas C2214 and U2210 are located in a hairpin loop of helix 79 in Domain V (Figure 2c). Thus, this is again a tertiary interaction joining distant regions of the molecule. Visual inspection revealed that the G:C trans base within this quartet motif can assume three different conformations while maintaining similar interactions with the additional bases, see below.

In addition to triplet and quartet interactions, we detected 310 instances where a structured water molecule is present nearly coplanar and within H-bond distance of freely accessible carbonyl and/or amino atoms of the G:C W:W trans pair. This was observed in the context of tRNA molecules, as well as of *E. coli* and *T. thermophilus* 23 S rRNA molecules. It is worth noting that the 310 instances we found is only a lower limit because it cannot be excluded that structured water molecules could have been simply omitted in some of the analyzed crystal structures. Moreover, we detected 269 instances where a phosphate oxygen atom is present nearly coplanar and within H-bond distance of accessible amino atoms of the G:C trans pair. Some of the interactions were found to be conserved, such as for instance the phosphate backbone of A750 in 23 S rRNA from *E. coli* that interacts with the G1271:C1615 base pair, where G1271 and C1615 are part of junction 26/47 and junction 49/51 in Domain III respectively, whereas A750 is present in the hairpin loop of helix 35 in Domain II. The oxygen of the phosphate backbone thus facilitates the interaction between Domain II and Domain III in 23 S rRNA molecule. Another recurrent motif is found in 23 S rRNA from *T. thermophilus*. It involves the interaction of the phosphate oxygen of U2473 with the G2529:C2475 base pair. C2475 and U2473 are located in the hairpin loop of helix 89 in Domain V, and G2529 in the Domain V helix 91 of the 23 S rRNA molecule. Several other interactions involving phosphate oxygens and joining distant-in-sequence regions are reported in the Supplemental Materials (Supplementary Table S1). Finally, we also detected 34 occurrences, in tRNAs and in *E. coli* and *T. thermophilus* 23 S rRNA molecules, where both a water molecule and a phosphate oxygen atom are within H-bond distance from the carbonyl and amino atoms, respectively, of the G:C trans pair.

A special case of higher order structure, involving a G:C W:W trans pair, is the binding of the preQ_1_ metabolite to the class II preQ_1_ riboswitch, whose new fold structure has been recently solved (35). preQ_1_, which is the final metabolite during the biosynthesis of the hypermodified nucleotide queuosine, bears a charged amino group on the C7 atom of the 7-deazaguanine core. The preQ_1_ binding pocket in this riboswitch resides in a three-way junction between three stems, see Figure 2d. There, preQ_1_ engages in a G:C W:W trans pair with conserved base C30, located in the J2-3 loop. Three additional H-bonds are contributed by U41, interacting with its Watson–Crick edge with the Sugar edge of preQ_1_. Mutation of C30 to U and of U41 to C caused a loss in affinity by a factor 46 and 90, respectively (35). Owing to the presence of a W:W trans pair, the binding of preQ1 to this riboswitch has also been included in our analysis.

**Figure 3.**
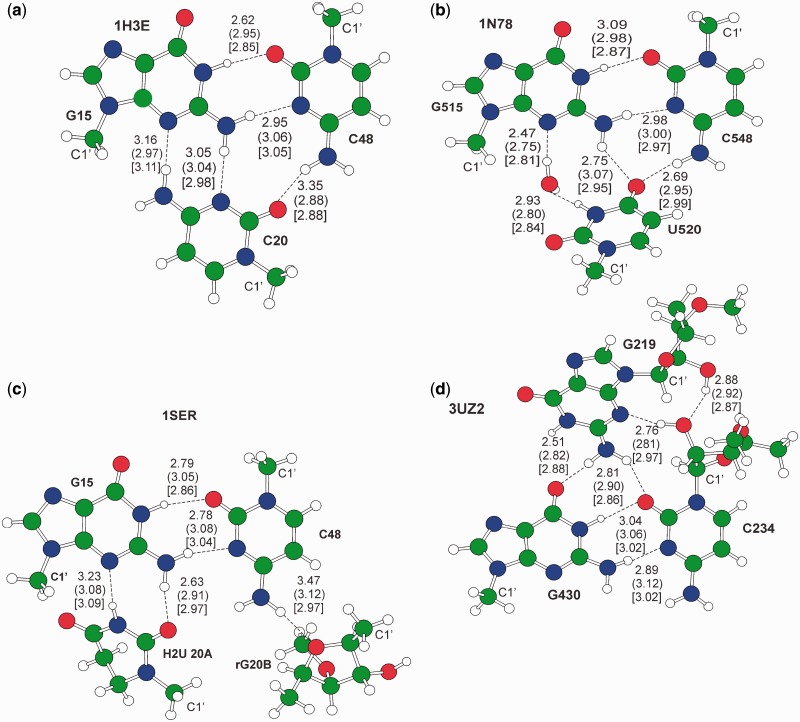
Gas phase optimized geometries of triplet motifs. H-bond distances between heavy atoms are reported in Å. Out of parentheses are the values in the crystal structure, in parentheses are the gas phase optimized structures, in square brackets values for the in water optimized geometries. The C1’ ribose atom is also labeled. Motifs (a–d) from PDBs 1H3E, 1N78, 1SER and 3UZ2, respectively.

**Figure 4.**
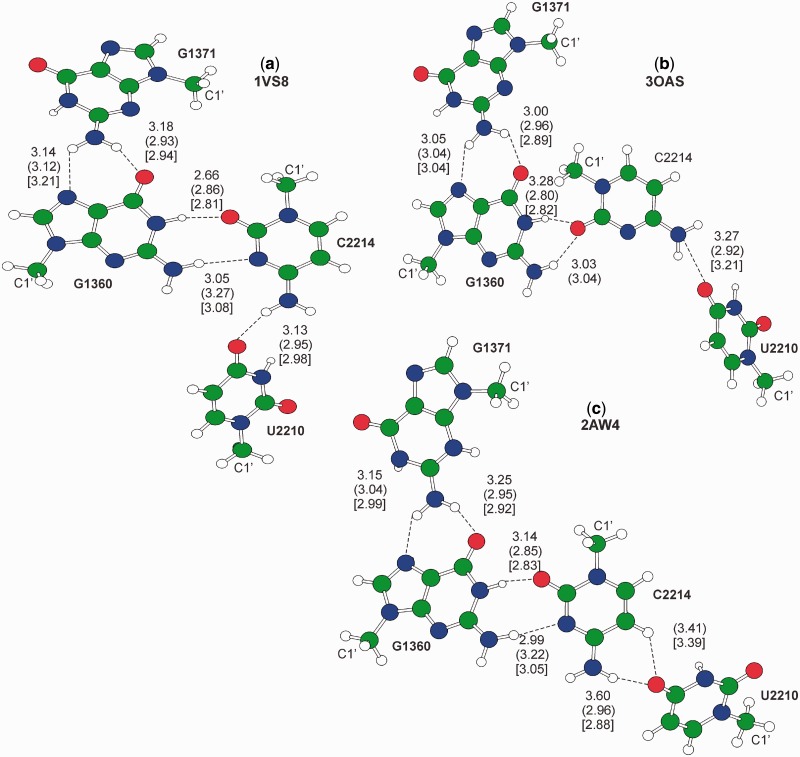
Gas phase optimized geometries of quartet motifs. H-bond distances between heavy atoms are reported in Å. Out of parentheses are the values in the crystal structure, in parentheses are the gas phase optimized structures, in square brackets values for the in water optimized geometries. The C1’ ribose atom is also labeled. Motifs (a–c) from PDBs 1VS8, 3OAS and 2AW4, respectively.

**Figure 5.**
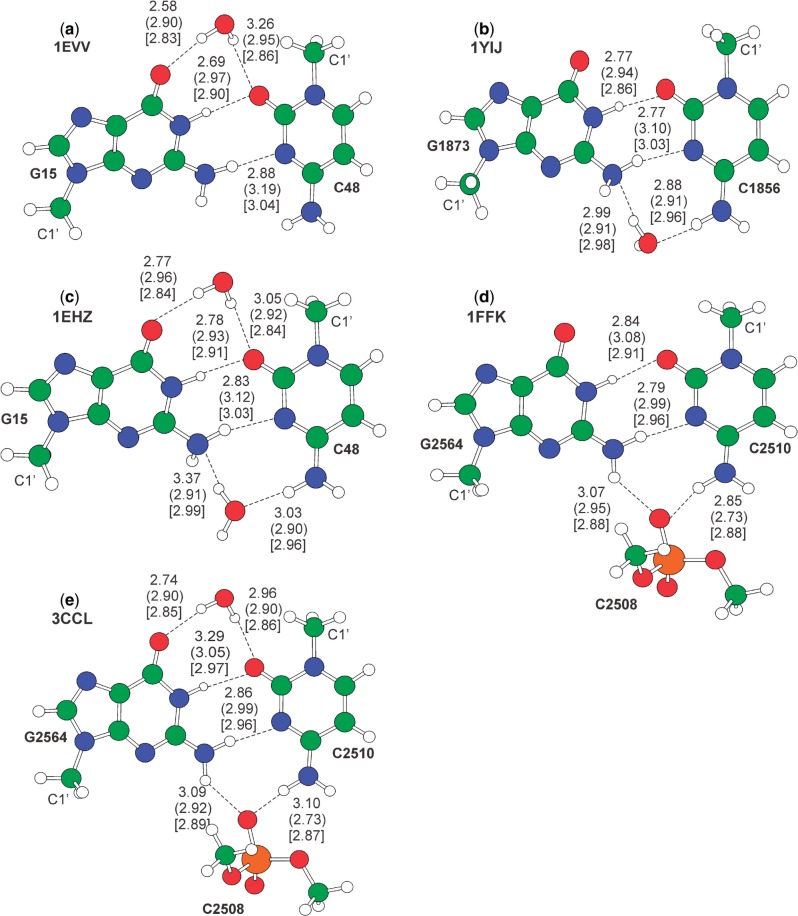
Gas phase optimized geometries of the G:C W:W trans pair in the presence of water and phosphate backbone. H-bond distances between heavy atoms are reported in Å. Out of parentheses are the values in the crystal structure, in parentheses are the gas phase optimized structures, in square brackets values for the in water optimized geometries. The C1’ ribose atom is also labeled. Motifs (a–e) from PDBs 1EVV, 1YIJ, 1EHZ, 1FFK and 3CCL, respectively.

**Figure 6.**
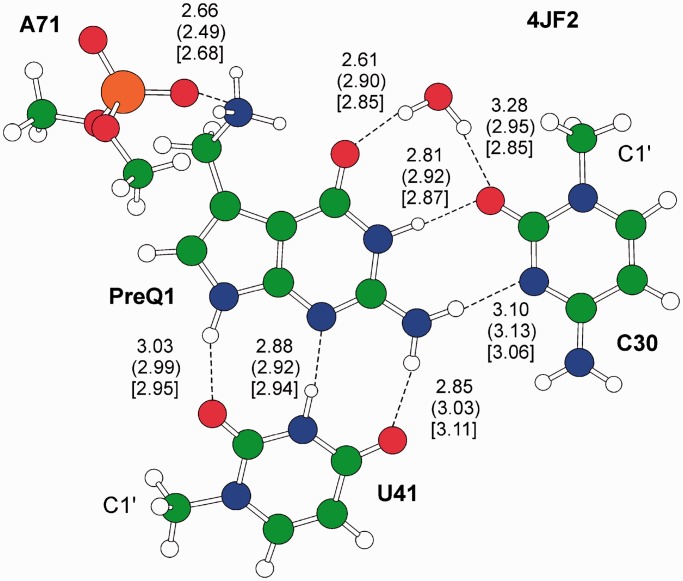
Gas phase optimized geometry of the H-bonding network in the preQ_1_ binding site, presenting a preQ_1_:C W:W trans morif. H-bond distances between heavy atoms are reported in Å. Out of parentheses are the values in the crystal structure, in parentheses are the gas phase optimized structures, in square brackets values for the in water optimized geometries. The C1’ ribose atom is also labeled.

Concluding this part, it is clear that our analysis revealed a spectrum of different strategies that RNA molecules exploit to stabilize the G:C trans base pairing geometry and maintain their tertiary structure. To have a better understanding of the contribution of these strategies to the stability of the G:C W:W trans pair, we performed high-level QM calculations on a selected representative for each experimentally observed structural motif. Table 1 summarizes the details about the investigated geometries and the reference structures in the wwPDB, while the interaction energy for both gas phase and solvent phase optimized geometries, computed as described in the ‘Materials and Methods’ section, are given in Table 2. The gas phase and in water optimized geometries are shown in Figures 3–6.

**Table 2.** Clamping energy, *E*_clamp_ and G:C interaction energy, *E*_GC_, in kcal/mol, for the representative of the systems reported in Table 1

Motif	E_clamp_ (Gas)	E_clamp_ (water)	E_GC_ (Gas)	E_GC_ (water)
GCC tWW/tSW	−24.9	−10.6	−14.4	−8.6
GCU tWW/Intermediate-O4(U)	−23.8	−10.6	−13.8	−8.5
GCH2U(rG) tWW/tSW	−20.9	−11.4	−13.8	−8.6
GrCrG tWW/cSS	−33.3	−16.4	−14.4	−8.2
GGCU(i) tHS/tWW/tWW	−21.1	−6.4	−14.7	−8.4
GGCU(ii) tHS/tWW/tWW	−20.2	−8.1	−18.3	−8.6
GGCU(iii) tHS/tWW/tWW	−23.4	−8.0	−14.9	−8.6
GC tWW/wc	−10.1	−5.5	−14.6	−8.3
GC tWW/wa	−12.2	−3.8	−14.8	−8.4
GC tWW/wcwa	−21.4	−8.1	−14.7	−6.2
GC tWW/OP1	−37.7	−7.2	−13.6	−8.2
GC tWW/OP1wc	−48.7	−12.9	−13.8	−8.0

As reference values, the interaction energy of the canonical G:C and A:U pairs, and of the Wobble G:U pair are −26.7, −14.0 and −16.2 kcal/mol in the gas phase, and −13.3, −8.1 and −8.0 kcal/mol in water, respectively.

### Quantum mechanical calculations

Details on the results of the QM calculations are given in the Supplementary Materials. In the following, only the main results are reported.

#### Triplets

The optimized geometry of the four triplet motifs we considered is shown in Figure 3. In addition to the overall structural description of the previous section, we notice here that analysis of the PDB structures evidenced that (i) in the GCU tWW/Intermediate triplet of Figure 3b, there is a structured water involved in H-bonds with N3(U) and N3(G), (ii) in the CGH2U(rG) tWW/tSW motif of Figure 3c, N4(C48) is H-bonded to the G20B ribose and (iii) in the GrCrG tWW/cSS motif of Figure 3d, there are two H-bonds involving the riboses of G219 and C234. Therefore, in the models used in the QM calculations, we included the above mentioned explicit water molecule and riboses. The optimized gas phase and in water geometries of the four motifs are similar to the experimental geometry, see Figure 3, with no sensible alteration of the crystallographically-observed H-bonding pattern. With a few exceptions, the difference in most of the H-bond distances is normally within 0.20 Å, which is standard in this kind of calculations. The explicit water molecule of the motif of Figure 3b retains its structural role, further confirmed by a gas phase optimization excluding the water molecule, which converged into a bifurcated geometry. Focusing on the motif of Figure 3c, a substantially similar geometry was found by replacing H2U with the unmodified U residue. In summary, the main message conveyed by this brief geometrical analysis is the overall consistency between the calculated and the experimental geometries in all the four cases of Figure 3, which indicates that the additional moieties act as a ‘clamp’ holding in place the G:C trans geometry. Moving to the interaction energy, it is worth noting the rather similar strength of the clamping interaction between the G:C trans motif and the additional moieties, *E*_clamp_, in the first three motifs of Figure 3, with values around −20 kcal/mol in the gas phase and −11 kcal/mol in water, see Table 2. The clearly larger *E*_clamp_ of G219 in the CGH2U(rG) tWW/tSW motif of Figure 3d, is owing to the additional two H-bonds involving the riboses of G219 and C234. Nevertheless, the main conclusion here is that the *E*_clamp_ is remarkable in magnitude, which explains the ability of the additional group to hold in place the G:C tWW geometry. As final remarks, the interaction energy between the water molecule and the triplet in the motif of Figure 3b is −17.8 and −8.2 kcal/mol in the gas phase and in water, which indicates that this water molecule is firmly held in place, while replacing the H2U residue of the motif of Figure 3c with the unmodified U residue results in an *E*_clamp_ only −0.7 kcal/mol larger.

#### Quartets

As mentioned above, the three quartet motifs we found correspond to three different conformations of the same GCCU motif, see Figure 4. The conformation of the motif of Figure 4a is the classical G:C W:W trans geometry, while the conformation of the motifs of both Figures 4b and c is bifurcated in the crystal structures. Gas phase and in water geometry optimization of the motif of Figure 4a resulted in a geometry similar to the experimental one, with differences in the H-bond distances within 0.20 Å, see Figure 4a. Differently, in water optimization of the motif of Figure 4b resulted in a rearrangement from the bifurcated G:C Ww/Bs trans geometry to the G:C W:W trans one, while the gas phase optimization resulted in a geometry similar to the experimental one, although with is a severe shortening of the N1-H(G) … O2(C) and N4-H(C) … O4(U) H-bonds by 0.48 Å and 0.35 Å, respectively. Finally, optimization of the motif of Figure 4c resulted in a shift from the bifurcated G:C Ww/Bw trans geometry to the G:C W:W trans geometry, both in the gas phase and in water. This indicates a noticeable flexibility of this motif, characterized by several geometrically close minima that can probably easily interconvert one to the other. As final remark, test calculations in which only one of the two additional G1371 and U2210 bases was included in the geometry optimization resulted in the starting C2214:G1360 W:W trans geometry to collapse into the bifurcated W:W Ww/Bs geometry, highlighting the concerted role played by G1371 and U2210, which basically tether C2214 and G1360 to the W:W trans geometry. Also in this case the overall *E*_clamp_ is high, around −20 and −8 kcal/mol in the gas phase and in water, respectively, and it is similar in the three conformations considered. This is consistent with the limited variation in the relative disposition of the two bases acting as a clamp. Indeed, comparison of the crystallographic structure of the three conformations indicates that the N2(G1371)-O4(U2210) distance is 11.5 Å in 1VOS, 12.7 Å in 3OAS and 11.9 Å in 2AW4, while the angle between the C2(G1371)-N2(G1371) and O4(U2210)-C4(U2210) bonds is 94.3°, 88.6° and 89.6° in the three conformations.

### Impact of ordered surrounding water molecules

From the analysis of the wwPDB we identified three representative cases, shown in Figure 5a–c, where the G:C trans pair presents H-bond interactions with water molecules, while presenting no additional H-bond with other bases. Considering the structural role of these water molecules, we treated them explicitly rather than embedding them into a continuum solvation model. Of course, in this case the calculated *E*_clamp_ can be considered as part of the solvation energy. The first case, GC tWW/wc (where ‘wc’ stays for water to carbonyl), corresponds to occurrences where a single water molecule is present nearly coplanar and within H-bond distance from the carbonyl groups of the G:C trans pair. The second case, GC tWW/wa (where ‘wa’ stays for water to amino), corresponds to a water molecule being nearly coplanar and within H-bond distance from the amino groups of the G:C trans pair. Finally, the third case, GC tWW/wcwa (where ‘wcwa’ stays for water to carbonyl plus water to amino), corresponds to two water molecules being contemporarily within H-bond distance and nearly coplanar with both the accessible carbonyl and the amino groups of the G:C base pair.

Gas phase and in water geometry optimization of the motifs of Figure 5a–c resulted in geometries clearly consistent with the experimental structures. The only exception was the gas phase optimization of the tWW/wa motif, with the water molecule rearranging to act as a H-bond donor for the amino N2(G), consistently with the known ability of the amino group to be a H-bond acceptor (21). Nevertheless, the main result here is that even a single water molecule, either on the carbonyl or the amino side, is enough to hold in place the W:W trans geometry, mimicking the structural role played by higher order structure formation. The clamping interaction energy *E*_clamp_ is estimated to be around −10 and −5 kcal/mol in gas phase and in water, respectively, in the tWW/wc and tWW/wa motifs. Not surprisingly, *E*_clamp_ in presence of two water molecules is approximately equal to the sum of the *E*_clamp_ of single water in the GC tWW/wc and GC tWW/wa systems.

### Impact of the phosphate backbone on the stability of G:C W:W trans pair

During the analysis of the wwPDB, we found several instances where a phosphate oxygen is coplanar and within hydrogen bonding distance from the amino N4(C) and N2(G) atoms, see Figure 5d. In a number of instances, a water molecule is additionally bridging the G:C pair from the carbonyl side, see Figure 5e. The optimized geometry of the selected model cases for these motifs indicate a H-bonding arrangement in good agreement with the experimental geometries, with differences in the H-bonding distances within 0.20 Å. Interestingly, optimization substantially improves the planarity of the G:C trans base pair geometry. The calculated clamping energy values in the gas phase are large, around 40–50 kcal/mol, values that are reduced to <10 kcal/mol in water. The large *E*_clamp_ value in the gas phase is of course consequence of the strong electrostatic interaction between the anionic phosphate and the GC base pair, an effect that is largely damped by the continuum solvation model in the in water calculations.

### The preQ1-II riboswitch binding motif

Optimization in the gas phase and in water of PreQ1 in its binding site inside the class II preQ_1_ riboswitch from *L.rhamnosus* results in a geometry that is in good agreement with the crystallographic structure (35), see Figure 6, although in the geometry optimization only the heavy atoms of U41 and of the phosphate of A71 were frozen, whereas the preQ_1_ moiety, the C30 and the structured water molecule were fully optimized. We did not include U31 in the calculations, although its O2 atom is engaged in an H-bond interaction with the NH_3_^+^ group of preQ_1_ because U31 is also engaged in a energetically relevant stacking interaction with preQ_1_, while this work is focused on H-bonds interactions only. Consistently with the discussion above, the single water molecule is able to preserve the W:W trans geometry of the preQ_1_:C motif. Nevertheless, we also optimized the geometry of the overall system without the water molecule, and the W:W trans preQ_1_:C motif was retained. This is probably owing to the positive –NH_3_^+^ group that, in analogy to what we found for the binding of a Mg^2+^ ion to the N7(G) atom, and similarly to the case of the archaeosine posttranscriptional modification (12), polarizes the electron density of the nearby O6(preQ_1_) atom, thus reducing the repulsive carbonyl–carbonyl interaction between the O6(G) and O2(C) atoms.

Moving to energies, we first calculated the energy associated with the building of the binding site of preQ_1_. To this end, we calculated the energy change associated with moving from infinite distance the A71 phosphate, U41, C30 and the structured water to the geometry they have when bound to the preQ_1_. In the gas phase and in water the interaction energy between these residues is −5.7 and −3.6 kcal/mol, indicating that assembling the binding site is a substantially thermoneutral process. The binding of the preQ_1_ in the assembled binding site amounts to −160.6 kcal/mol in the gas phase, a value reduced to −28.7 kcal/mol in water. The large value in the gas phase is consequence of the unscreened electrostatic interaction between the A71 phosphate and the nearby NH_3_^+^ group of preQ_1_. Focusing on the more realistic −28.7 kcal/mol value, we decomposed it into the various components, by removing one single piece (either A71, U41 or C30 together with the water molecule) from the structure reported in Figure 6, and by calculating the interaction energy between the removed piece and the remaining of the structure. Interestingly, the electrostatic preQ_1_/A71 phosphate interaction amounts to −8.6 kcal/mol only, and thus it is similar to the preQ_1_/C30 interaction, −8.8 kcal/mol, and is surpassed by the preQ_1_/U41 interaction, −11.5 kcal/mol. These findings are consistent with the experimental data, showing a loss in affinity by a factor of 46 and 90, on mutation of C30 to U and of U41 to C, respectively (35). The preQ_1_/water interaction amounts to additional −4.7 kcal/mol, so that the overall preQ_1_/(C30+water) interaction amounts to the remarkable value of −13.6 kcal/mol, thus giving the strongest contribution to the preQ_1_ binding.

## DISCUSSION

Looking at the structure of the G:C W:W trans pair, it is evident that the exocyclic amino N4(C) and N2(G) atoms and the carbonyl O2(C) and O6(G) atoms are freely accessible for further H-bonding interactions. This is particularly relevant owing to the intrinsic instability of the G:C W:W trans geometry (12,60) and to its occurrence in higher level interactions often joining together different structural motifs in functional RNAs. We observed and characterized higher order geometries involving the G:C W:W trans pair, which maintain the tertiary structure of tRNA molecules and of ribosomal RNAs, and even contribute to the metabolite recognition in riboswitches. We have previously shown that posttranscriptional modification or divalent metal ions binding to the N7 atom of the guanine stabilizes the geometry of the G:C W:W trans base pair (12,14). Here we have shown that other environmental factors, such as additional bases, phosphate backbone atoms and even structured water molecules can help to hold the G:C W:W trans geometry in place, by functioning as a clamp, with a variety of possible solutions spanning from simpler motifs, with a single additional base, phosphate or water molecule acting as a clamp (see Figures 3a and 5a, b, d), to more complex structures where the clamping is provided by the concerted action of two bases (see Figure 4), two water molecules (Figure 5c) or one base and a ribose (Figure 3c). Of particular interest is the highly concerted action of a base and of a water molecule (Figure 3b), with the water molecule acting as a bridge between the G:C pair and the additional base. In all the cases, the clamping interactions seem to balance the carbonyl–carbonyl and amino–amino repulsion of the G:C W:W trans base pair, thus avoiding its geometrical rearrangement to the bifurcated G:C Ww/Bs trans geometry, having comparable interaction energy but being highly nonisosteric. The high involvement of water molecules in keeping together the G:C W:W trans geometry is not surprising, as it is well known that folding properties and conformation of nucleic acids are significantly affected by solvation (61–64) and many of the reported base pairing geometries in RNA structures are found to be closely associated with bound water molecules with reduced mobility (18,65).

Focusing on energy, the variability in the possible structural solutions results in a variability of the calculated clamping energy, spanning in water, treated as a continuum, the −4 to roughly −12 kcal/mol range. The weakest clamping is provided by a single explicit water molecule, while the strongest is provided by the concerted action of more groups, such as in the GrCrG tWW/cSS and GC tWW/PO1wc systems. Consistently with the building block nature of stabilizing interactions in nucleic acids, the total clamping energy can be estimated from the clamping energy of simpler motifs. For example, the clamping energy of a single water molecule H-bonded to the carbonyl or to the amino side (Figure 5a and b), is roughly −4/−5 kcal/mol, while the clamping energy of two water molecules, one on the amino and the other on the carbonyl side, amounts to roughly −8 kcal/mol. Similarly, the clamping provided by a single phosphate (Figure 5d) amounts to roughly −7 kcal/mol, which, summed to the roughly −5 kcal/mol for the clamping provided by a single water molecule, allows to predict with reasonable accuracy the total clamping energy in case of a concerted clamping of a phosphate and a water molecule, −12.9 kcal/mol in 3CCL (Figure 5e). This indicates that the values we have calculated for the single clamping interactions can be used to estimate the strength of the interaction in different structures beyond those discussed in the present article. Furthermore, analysis of the G:C base pairing energy in the examined cases indicates that it is remarkably high in all the environments considered, with an average value of −8.2 ± 0.7 kcal/mol, and comparable with that of the canonical G:C and A:U W:W cis base pairs (having a base pairing energy in water of −13.3 and −8.1 kcal/mol, respectively). This confirms again that the weakness of the G:C W:W trans pair is not in small interaction energy between the two bases. In fact, once held in place, it is stabilized by two H-bonds as the canonical A:U W:W cis base pair. Rather, its weakness is in its geometrical instability, with the tendency to fall into the similarly stable bifurcated G:C Ww/Bs geometry. This can be consistent with the occurrence of the G:C W:W trans geometry in fundamental interactions contributing to the overall folding of nucleic acids, or in the recognition of metabolites in riboswitches. In case the G:C W:W trans geometry has to be rigidly held in place in the functional RNA, other nucleotides or water molecules can in fact stabilize its geometry. However, if the interaction connects sections of the system that have to undergo functional rearrangements, the possibility to easily switch to a different base pairing geometry can also be strategical.

## CONCLUSIONS

In this work, we searched for the occurrence of the G:C W:W trans geometry in RNA structures, and we analyzed the resulting geometries in terms of additional H-bonding interactions with other moieties of RNA (bases, phosphate, sugar), as well as with structured water molecules. Our structural database analysis revealed the importance of additional H-bonds from the surrounding structural context, as they occur in ∼70% of the all G:C trans base pairs observed in the wwPDB. The frequency that these additional interactions occur with suggests that they are probably fundamental to the RNA folding and functioning. The main conclusions of our work can thus be summarized as follows:

- The G:C W:W trans geometry is a recurrent structure in functional RNAs, with a privileged role in participating in higher order motifs, often joining together different structural domains.
- A single base or even a single water molecule, acting as a H-bonding clamp, is able to give structural stability to the G:C W:W trans geometry. Clamping can occur on the exocyclic N2(G)/N4(C) amino side as well as on the O6(G)/O2(C) carbonyl side, and often involves both the amino and the carbonyl sides.
- The clamping energy can span the large window between roughly −5 and −15 kcal/mol in water, the weakest value being associated to the clamping operated by a single water molecule. These clamping energies are close in magnitude to the H-bonding energy of the classical A:U and G:C WC base pairs.
- The preQ_1_:C W:W trans interaction, in the binding site of a preQ_1_ class II riboswitch, is not negligible, contributing ∼25% of the total H-bonding energy between preQ_1_ and the riboswitch binding site. When also the clamping water molecule is considered, it provides the strongest H-bond contribution to the preQ_1_ binding.

In conclusion, our bioinformatics and QMs analysis indicated that the G:C W:W trans base pair, stabilized by H-bonding with other entities, contributes fundamental tertiary interactions in the context of different RNA molecules, helping to maintain their fold and hence functionality. Interestingly, its stabilization can be achieved in a number of building-block type structures, spanning a large energy window, thus offering evolution a large variety of structural solutions from which to select to achieve functionality.

## SUPPLEMENTARY DATA

Supplementary Data are available at NAR Online.

## FUNDING

Funding for open access charge: Baseline research funds from KAUST.

*Conflict of interest statement*. None declared.

## Supplementary Material

Supplementary Data
