# Supplementary material for: Higher order structural effects stabilizing the reverse Watson–Crick Guanine-Cytosine base pair in functional RNAs
Source: Nucleic Acids Res. 2013 Oct 8;42(2):714–26. doi: 10.1093/nar/gkt800 (PMC3902895; doi:10.1093/nar/gkt800)
Supplement: Supplementary Data [file supp_42_2_714__index.html]

Higher order structural effects stabilizing the reverse Watson–Crick Guanine-Cytosine base pair in functional RNAs — Higher order structural effects stabilizing the reverse Watson–Crick Guanine-Cytosine base pair in functional RNAs — Supplementary Data 

# Higher order structural effects stabilizing the reverse Watson–Crick Guanine-Cytosine base pair in functional RNAs

## Supplementary Data

files

**Files in this Data Supplement:**

- Supplementary Data - pdf file
