# Supplementary material for: Higher order structural effects stabilizing the reverse Watson–Crick Guanine-Cytosine base pair in functional RNAs
Source: Nucleic Acids Res. 2013 Oct 8;42(2):714–26. doi: 10.1093/nar/gkt800 (PMC3902895; doi:10.1093/nar/gkt800)
Supplement: Supplementary Data [file supp_gkt800_nar-01456-r-2013-File002.pdf]

Supporting Information for

Higher Order Structural Effects Stabilizing the Reverse Watson-Crick Guanine-Cytosine Base Pair in Functional RNAs

Mohit Chawla,[1] Safwat Abdel-Azeim,[1] Romina Oliva[2,*] and Luigi Cavallo[1,*]

[1] Kaust Catalysis Center, Physical Sciences and Engineering Division, King Abdullah University of Science and Technology, Thuwal 23955-6900, Saudi Arabia. [2] Department of Applied Sciences, University of Naples "Parthenope", Centro Direzionale Isola C4, I-80143, Naples, Italy

Detailed results of quantum mechanical calculations

GCC tWW/tSW triplet. In this triplet, the additional cytosine uses its 'Watson-Crick' edge to interact with both the 'Sugar edge' of the guanine and the exocyclic N4 of the cytosine of the G:C trans motif, giving the following H bonds: N4-H(C)…N3(G), N2-H(G)…N3(C), and N2-H(C)…O2(C), see Figure 3a. The starting geometry for the computational studies is that observed in the $tRNA^{Tyr}$ structure from *T. thermophilus* (PDB ID: 1H3E). The optimized gas phase and in water geometries are quite similar to the experimental geometry, with no alteration of the X-ray observed H-bonding pattern and differences in most of the H-bond distances within 0.10 Å, see Figure 3a. Larger deviation is found for the N1-H(G)…O2(C) H-bond of the G:C W:W trans motif, which is stretched by 0.33 Å and 0.23 Å in the gas phase and in the water optimized geometries, whereas the H-bond N4-H(C)….O2(C) is reduced by 0.47 Å in both the gas phase and in water optimized geometries. Looking at the triplet geometry, the additional cytosine interacting with both bases of the G:C W:W trans motif appears to be acting as a "clamp" holding in place the G:C trans geometry. The strength of the clamping interaction between the G:C trans motif and the additional cytosine, E_{clamp}, is -24.9 kcal/mol in the gas phase and -10.6

kcal/mol in water, see Table 2, indicating that the cytosine is firmly bound to the G:C trans base pair. The interaction energy between the guanine and the cytosine in the G:C pair, E_{GC}, is -14.4 and -8.6 kcal/mol in the gas-phase and water, respectively, see Table 2. The clamping interaction is dominated by the Hartree-Fock component, which constitutes 73% of total interaction energy, indicating that the H-bonding interaction energy, consistently with literature, is mainly electrostatic in nature.

GCU tWW/Intermediate O4(U) triplet. The crystal structure of $tRNA^{Glu}$ from *T. thermophilus* was taken as a reference system for this motif (PDB ID: 1N78). The crystal geometry exhibits a H-bond between O4(U) and the exocyclic amino groups N4(C) and N2(G) of the G:C trans base pair. In addition, analysis of the PDB structure revealed the presence of a structured water involved in H-bonds with N3(U) and N3(G), and thus this water molecule was included in the calculations. The optimized structures are quite close to the experimental geometry. The larger deviations involve the N4-H(C)….O4(U), N2-H(G)….O4(C) and O-H(w)…N3(G) H-bond distances, which are elongated by 0.26/0.32/0.28 Å in the gas phase and by 0.31/0.20/0.34 Å in water, see Figure 3b. The explicit water molecule retains its structural role, by bridging the U20 and G15 residues. The structural role of this water molecule was confirmed by a gas-phase optimization of the triplet while excluding the water molecule, which resulted in a severely distorted non-coplanar geometry with formation of an artificial contact between N3(U) and N3(G). Thus, it seems that U20 and the water molecule act together as a clamp, aiding the geometric stability of G:C trans base pair motif. The computed water+U….G:C clamping interaction energy E_{clamp} is -23.8 and -10.6 kcal/mol in the gas phase and in water, respectively, while the G:C interaction energy is -13.8 and -8.5 kcal/mol in the gas phase and in water. Finally, the interaction energy between the water molecule and the triplet is -17.8 and -8.2 kcal/mol in the gas phase and in water, which indicates that the water molecule is firmly held in place.

CGH2U(rG) tWW/tSW Motif. This motif is only observed in the crystal structure of $tRNA^{Ser}$ from *T. thermophilus,* that was thus used as reference (PDB ID: 1SER). It involves a 5,6-dihydro-U residue, resulting from the post transcriptional modification of uracil, which interacts using its 'Watson-Crick' edge with the 'sugar edge' of the Guanine in the G:C W:W trans base pair, giving

the following H-bonds: N3-H(H2U)…N3(G) and N2(G)-H…O2(H2U). Gas phase optimization of the triplet motif leads to the conformational collapse of the G:C base pair into the bifurcated Ww/Bs trans geometry. Careful examination of the crystal structure revealed the presence of the ribose atom O4'(G20B) within H-bonding distance (3.47 Å) from N4(C48). Thus, ribose of G20B residue was also included in the calculations. Since H2U20A and the ribose of G20B are not covalently bonded in the model used for the QM calculations, their heavy atoms were frozen to the crystallographic position to preventing unrealistic conformational rearrangements of these groups.

Inclusion of the G20B ribose in the model resulted in the optimized geometries of the G:C pair well reproducing the experimental structure, with deviations in the H-bond distances smaller than 0.30 Å, see Figure 3c . This indicates that 5,6-di-hydro-U and the G20B ribose are acting in a concerted manner as a clamp that stabilizes the G:C trans geometry. The gas phase and in water strength of the E_{clamp} between H2U plus the G20 ribose and the G:C pair are -20.9 and -11.4 kcal/mol, respectively, while the G:C base pair energy is -13.8 and -8.6 kcal/mol in gas phase and in water respectively. To gain insight into the possible effect of the chemical modification of H2U on the geometry and stability of the G:C pair, we also examined the GCUr(G) geometry in the gas-phase by replacing H2U with the unmodified U residue. Inclusion of the unmodified residue has negligible effect on the H-bond distances, with differences less than 0.20 Å relative to the optimized geometry of the structure including the H2U residue, and with a E_{clamp} only -0.7 kcal/mol larger. This similar stabilizing behavior can be understood in terms of the involvement of the 'Watson-Crick' edge of the U base in the pairing interaction, where the H-bonding network is similar for the U and 5,6-di-hydro-U residues.

GrCrG tWW/cSS Motif. The instance selected as reference for this motif is G430:C234:G219 from 23S rRNA of *T. thermophilus.* In it, the G219 uses its amino group to interact with the O2 atom of C234 and with the O6 atom of G430, giving the following H-bonds: N2-H(G219)…O2(C234) and N2-H(G219)…O6(G430). In addition to that, it interacts with its 'Sugar edge' with the ribose of C234, giving the O2'-H(C234)…N3(G219) H-bond, while its ribose H-bonds to the C234 ribose, O2'-H(G219)…O2'(C234). Gas-phase optimization of this motif

without inclusion of the G219 and C234 ribose moieties resulted in a geometry inconsistent with the experimental structure, with the G:C pair reaching a Ww/Bs geometry. Inclusion of both the above ribose moieties resulted in a geometry similar to the crystallographic one, with the H-bonding pattern deviation of no more than 0.30 Å from the experimental values, see Figure 3d. The E_{clamp} of G219 with the G430:C214 W:W trans pair was estimated to be as high as -33.3 and -16.4 kcal/mol in the gas phase and in water, respectively. This high interaction energy can be explaining by considering that G219 is involved in four H-bonds with the G:C W:W trans pair. Finally, the interaction energy between G and C bases of the G:C pair is -14.4 and -8.2 kcal/mol in the gas phase and in water, respectively.

GGCU tHS/tWW/tWW Motif. In this quartet, the additional guanine uses its N2 atom to give N2(G)-H…N7(G) and N2(G)-H…O6(G) H-bonds with the 'Hoogsteen edge' of the guanine in the G:C trans pair, while U utilizes its O4 atom to form N4-H(C)…O4(U) H-bond with the cytosine. However, after visual inspection of all these motifs in the available dataset, it turned out that three different conformations of the G:C trans base pair exist, with a similar surrounding interaction pattern within the GGCU motif. The first conformation is the classical G:C W:W trans geometry and we found 17 occurrence of it, see Table 1. The second conformation is the bifurcated Ww/Bs trans geometry that we already discussed, and we found 5 occurrences of this geometry. The third conformation is again a bifurcated one, but in it, instead of the O2(C), it is N3(C) that is involved in the bifurcated H-bonds with N1- and N2-H(G). It can be classified as G:C Ww/Bw trans geometry. We observed 8 occurrences of this third conformation. To understand the role of the quartet formation on the stability of the G:C trans pair, we optimized a representative of all the three conformations (G1360:C2214:G1371:U2210 from 23S rRNA of *E. coli*, PDB ID: 1VS8, 3OAS, 2AW4, respectively), by freezing the coordinates of the heavy atoms of the additional G and U residues to prevent rearrangements unfeasible in the 23S environment.

For the first representative conformation considered, with a G:C W:W trans geometry, the gas phase and in water geometry optimizations resulted in a geometry similar to the experimental one, with differences in the H-bond distances within 0.20 Å, see Figure 4a. The gas phase and in

water E_{clamp}, -21.1 and -6.4 kcal/mol, is quite high, and the geometry is consistent that of experimental with the formation of N2-H(G)…N7(G), N2-H(G)…O6(G) and N4-H(C)…O4(U) H-bonds between the G:C pair and the other bases. The interaction energy of the G:C pair instead amounts to –14.7 and –8.4 kcal/mol in the gas-phase and in water. For the second conformation, with a G:C Ww/Bs trans geometry, the gas phase optimized geometry is similar to the experimental one in the H-bonding pattern, although there is a quite severe shortening of the N1-H(G)…O2(C), and N4-H(C)…O4(U) H-bonds by 0.48 Å and 0.35 Å, respectively, see Figure 4b. In contrast, the in water optimization resulted in a rearrangement from the bifurcated G:C Ww/Bs trans geometry to the G:C W:W trans one. This is followed by the formation of an additional weak H-bond, C5(C)-H…O4(U). Also in this case the gas phase and in water E_{clamp} is quite high, -20.2 and -8.1 kcal/mol. The interaction energy of the G:C pair instead amounts to –18.3 and –9.8 kcal/mol in the gas-phase and in water. Finally, for the third conformation, both the gas phase and in water optimizations resulted in a shift from the bifurcated G:C Ww/Bw trans geometry to the G:C W:W trans geometry, with the additional formation of a H-bond between C5-H(C) and O4(U) atoms, see Figure 4c. Interestingly, test calculations in which only one base between G1371 and U2210 was included in the geometry optimization resulted in the starting C2214:G1360 W:W trans geometry to collapse into the bifurcated W:W Ww/Bs geometry, highlighting the concerted role played by G1371 and U2210, which basically tether C2214 and G1360 to the W:W trans geometry.

The E_{clamp} in the case of 2AW4, -23.4 and -8.0 kcal/mol in the gas-phase and in water, is the strongest among the three GGCU quartets. Comparison of the crystallographic structure of the three conformations indicates that the N2(G1371)-O4(U2210) distance is 11.5 Å in 1VOS, 12.7 Å in 3OAS, and 11.9 Å in 2AW4, while the angle between the C2(G1371)-N2(G1371) and O4(U2210)-C4(U2210) bonds is 94.3°, 88.6° and 89.6° in the three conformations. This highlights the impact that limited variations in the relative disposition of the two bases acting as a clamp can have on the preferred structure and the energetics of the central G:C base pair.

Impact of ordered surrounding water molecules. From the crystal structure database analysis, we found 160 instances where a single water molecule is present nearly coplanar and within H-

bond distance from the carbonyl groups of the G:C trans pair, specifically from the O6(G) and O2(C) atoms, see Table 1. We will call them in the following: GC tWW/wc (where 'wc' stays for water to carbonyl). Furthermore, we found 82 occurrences of a water molecule nearly coplanar and within H-bond distance from the amino groups of the G:C trans pair, specifically from N2(G) and N4(C). We will call them in the following: GC tWW/wa (where 'wa' stays for water to amino). Finally, we found 68 occurrences where two water molecules are contemporarily observed within H-bond distance and nearly co-planar with both the accessible carbonyl and the amino groups of the G:C base pair. We will call them in the following: GC tWW/wcwa (where 'wcwa' stays for water to carbonyl plus water to amino). Thus, for the subsequent QM analysis, three model systems were prepared by extracting the coordinates from the highest resolutions crystal structures corresponding to the three systems, GC tWW/wc, GC tWW/wa and GC tWW/wcwa.

GC tWW/wc motif. In this motif, a water molecule is acting as a donor simultaneously for both the O6(G) and O2(C) atoms of the G:C trans geometry, see Figure 5a. The fully relaxed geometries obtained from both the gas phase and in water optimizations are consistent with that of the crystal structure geometry. However, as compared to experimental geometry, the O-H(w)…O2(C) H-bond is shortened by 0.31 Å and 0.40 Å, the O-H(w)…O6(G) H-bond is elongated by 0.32 Å and 0.25 Å, and the N2-H(G)…N3(C) H-bond is elongated by 0.31 Å and 0.16 Å in gas phase and in water optimized geometries, respectively. Looking at the geometry, it is apparent that even a single water molecule is able to held in place the W:W trans geometry, mimicking the structural role played by higher order structure formation. The clamping interaction energy E_{clamp} is estimated to be -10.1 and -5.5 kcal/mol in gas phase and in water, respectively, while the interaction energy E_{GC} between the G and C bases is -14.6 and -8.3 kcal/mol in gas phase and in water, respectively.

GC tWW/wa motif. In this motif, a water molecule is interacting with the amino groups of the G:C W:W trans pair, specifically with the N2(G) and N4(C) atoms, see Figure 5b. In contrast to the in water optimization, where the oxygen atom of the water molecule is acting as an acceptor for both the accessible N2(G) and N4(C) donor atoms, the gas phase optimization

converged into a geometry with the following H-bonds: N4-H(C)…O(w) and O-H(w)…N2(G), where the amino atoms act as donor and acceptor, respectively, consistently with the known ability of the amino group to be a H-bond acceptor (21). Despite of this difference in the orientation of the water molecule, both the gas phase and in water optimization converged in a geometry that is quite similar to the experimental one. Nevertheless, the main point here is that a single water molecule interacting with the amino groups of both the G and C bases is again able to stabilize the W:W trans geometry. In water, the E_{clamp} for this system is slightly lower than that calculated for the GC tWW/wc one (-3.8 vs. 5.5 kcal/mol), while in the gas phase it is -2.1 kcal higher (-12.2 vs. 10.1 kcal/mol). Finally, the interaction between the G and C bases is -14.8 and -8.4 kcal/mol in gas phase and in water, respectively.

GC tWW/wcwa motif. At this point, it is not surprising that the geometry optimization of the G:C W:W trans pair in the presence of two water molecules, one on the carbonyl and the other on the amino side, results in a geometry similar with the experimental ones, see Figure 5c. Interestingly, the H-bonding distances in the gas phase and in water optimized geometries are highly consistent (H-bond distances within 0.01 Å). However, as compared to experimental geometry, the O(w)-H…O6(G) and O(w)-H…N2(G) H-bond is elongated by roughly 0.19 Å, while the O(w)-H…O2(C) H-bond is shortened by 0.21 Å, the N1(G)-H…O2(C) H-bond is shortened by 0.38 Å, and the O(w)H…N2(C) H-bond is shortened by 0.13 Å in the gas-phase optimized geometries. The clamping energy of the two water molecules on the G:C W:W trans pair is estimated to be -21.4 and -8.1 kcal/mol in the gas-phase and in water respectively. Not surprisingly, E_{clamp} in presence of two water molecules is approximately equal to the sum of the E_{clamp} of single water in the GC tWW/wc and GC tWW/wa systems. Finally, the interaction energy between the G and C bases is -14.7 and -6.2 kcal/mol in gas phase and in water, respectively.

Impact of the phosphate backbone on the stability of G:C W:W trans pair. Our analysis revealed 269 occurrences in the wwPDB, where a phosphate oxygen is found to be coplanar and within hydrogen bonding distance from the amino N4(C) and N2(G) atoms. To model this structural motif we optimized the G:C W:W trans pair in presence of the phosphate group,

using the G2564:C2510 base pair interacting with phosphate of C2508 residue, see Table 1. Of course, in this case the overall system bears a negative charge corresponding to the anionic phosphate moiety. The H-bonding arrangement in the gas phase and in water optimized geometries is in good agreement with that of the experimental geometry, with differences in the H-bonding distances within 0.20 Å, see Figure 5d. Interestingly, the gas phase and in water optimizations substantially improve the planarity of the G:C trans base pair geometry. The calculated clamping energy values are -37.7 and -7.2 kcal/mol in gas phase and in water, respectively. The large E_{clamp} value in the gas-phase is of course consequence of the strong electrostatic interaction between the anionic phosphate and the GC base pair, an effect that is largely damped by the continuum solvation model in the in water calculations. The interaction energy between G and C in the trans pair is -13.6 and -8.2 kcal/mol in gas phase and in water, respectively.

We also found 34 occurrences of the above geometry, with additional H-bonds between the carbonyl O2(C) and O6(G) atoms with a water molecule (similarly to the G:C tWW/wa system). This system was also subjected to QM calculations (see Figure 5e). The H-bonds pattern in the gas phase and in water optimized geometries is consistent with that of the experimental structure. The calculated clamping energy is as high as -48.7 and -12.9 kcal/mol in the gas-phase and in water. Finally, the interaction energy between G and C in the trans pair is –13.8 and -8.0 kcal/mol in gas phase and in water, respectively.

Table S1. Complete list of the occurrences in PDB structures of G:C trans base pairs involved in higher order motifs.

	PDB	Ch_ID	G	C	additional moieties
**GCC tWW/tSW Triplet**					
	**PDB**	**Ch_ID**	**G**	**C**	**additional moieties**
1	1h3e	B	15	48	C20
2	1wz2	C	915	960	C922
3	1wz2	D	915	960	C922
4	2der	C	15	47	C20
5	2der	D	15	47	C20
6	2det	C	15	47	C20
7	2deu	C	15	47	C20
8	2zzm	B	15	48	C20
9	2deu	D	15	47	C20
**GCU tWW/Intermediate-O4(U) Triplet**					
	**PDB**	**Ch_ID**	**G**	**C**	**additional moieties**
1	1g59	B	515	548	U519
2	1g59	D	515	548	U519
3	2cv1	C	515	548	U520
4	2cv1	D	515	548	U520
5	2cv2	C	515	548	U520
6	2cv2	D	515	548	U520
7	1n77	C	515	548	U520
8	1n77	D	515	548	U520
9	1n78	C	515	548	U520
10	1n78	D	515	548	U520
**GCH2U tWW/tSW Triplet**					
	**PDB**	**Ch_ID**	**G**	**C**	**additional moieties**
1	1ser	B	15	48	H2U 20A
**GCG tWW/cSS Triplet**					
	**PDB**	**Ch_ID**	**G**	**C**	**additional moieties**
1	2j01	A	234	430	G219
2	2j03	A	234	430	G219
3	2v47	A	234	430	G219
4	2v49	A	234	430	G219
5	2wdi	A	234	430	G219
6	2wdj	A	234	430	G219
7	2wdl	A	234	430	G219
8	2wdn	A	234	430	G219
9	2wh2	A	234	430	G219
10	2wh4	A	234	430	G219
11	2x9s	A	234	430	G219
12	2x9u	A	234	430	G219
13	2xg0	A	234	430	G219
14	2xg2	A	234	430	G219
15	2xqe	A	234	430	G219
16	2y0v	A	234	430	G219
17	2y0x	A	234	430	G219
18	2y0z	A	234	430	G219

19	2y11	A	234	430	G219
20	2y13	A	234	430	G219
21	2y15	A	234	430	G219
22	2y17	A	234	430	G219
23	2y19	A	234	430	G219
24	3d5b	A	234	430	G219
25	3d5d	A	234	430	G219
26	3f1f	A	234	430	G219
27	3f1h	A	234	430	G219
28	3hux	A	234	430	G219
29	3huz	A	234	430	G219
30	3i8f	A	234	430	G219
31	3i8i	A	234	430	G219
32	3i9c	A	234	430	G219
33	3i9e	A	234	430	G219
34	3kir	A	234	430	G219
35	3kit	A	234	430	G219
36	3kni	A	234	430	G219
37	3knk	A	234	430	G219
38	3knm	A	234	430	G219
39	3kno	A	234	430	G219
40	3oh5	A	234	430	G219
41	3oh7	A	234	430	G219
42	3ohj	A	234	430	G219
43	3ohk	A	234	430	G219
44	3ohz	A	234	430	G219
45	3oi1	A	234	430	G219
46	3oi3	A	234	430	G219
47	3oi5	A	234	430	G219
48	3pyo	A	234	430	G219
49	3pyr	A	234	430	G219
50	3pyt	A	234	430	G219
51	3pyv	A	234	430	G219
52	3tve	A	234	430	G219
53	3tvh	A	234	430	G219
54	3uxq	A	234	430	G219
55	3tvh	A	234	430	G219
56	3uxq	A	234	430	G219
57	3uxr	A	234	430	G219
58	3uye	A	234	430	G219
59	3uyg	A	234	430	G219
60	3uz1	A	234	430	G219
61	3uz2	A	234	430	G219
62	3uz8	A	234	430	G219
63	3uz9	A	234	430	G219
64	3uzf	A	234	430	G219
65	3uzh	A	224	458	G209
66	3uzk	A	234	430	G219
67	3uzn	A	234	430	G219
68	3v23	A	234	430	G219
69	3v25	A	234	430	G219

70	3v27	A	234	430	G219
71	3v29	A	234	430	G219
72	4abs	A	234	430	G219
73	4dha	A	234	430	G219
74	4dhc	A	234	430	G219

GGCU tHS/tWW/tWW Quartet

	PDB	Ch_ID	G	C	additional moieties
1	1vs6	B	1360	2214	G1371,U2210
2	1vs8	B	1360	2214	G1371,U2210
3	2aw4	B	1360	2214	G1371,U2210
4	2awb	B	1360	2214	G1371,U2210
5	2i2t	B	1360	2214	G1371,U2210
6	2i2v	B	1360	2214	G1371,U2210
7	2qam	B	1360	2214	G1371,U2210
8	2qao	B	1360	2214	G1371,U2210
9	2qbe	B	1360	2214	G1371,U2210
10	2qbg	B	1360	2214	G1371,U2210
11	2qoz	B	1360	2214	G1371,U2210
12	2qp1	B	1360	2214	G1371,U2210
13	3df2	B	1360	2214	G1371,U2210
14	3df4	B	1360	2214	G1371,U2210
15	3i1n	A	1360	2214	G1371,U2210
16	3i1p	A	1360	2214	G1371,U2210
17	3oas	A	1360	2214	G1371,U2210
18	3oat	A	1360	2214	G1371,U2210
19	3ofc	A	1360	2214	G1371,U2210
20	3ofd	A	1360	2214	G1371,U2210
21	3ofq	A	1360	2214	G1371,U2210
22	3ofr	A	1360	2214	G1371,U2210
23	3ofz	A	1360	2214	G1371,U2210
24	3og0	A	1360	2214	G1371,U2210
25	3orb	A	1360	2214	G1371,U2210
26	3r8s	A	1360	2214	G1371,U2210
27	3r8t	A	1360	2214	G1371,U2210
28	3sgf	A	1360	2214	G1371,U2210
29	4gar	A	1360	2214	G1371,U2210
30	4gau	A	1360	2214	G1371,U2210

GC WW trans/wc

	PDB	Ch_ID	G	C	additional moieties
1	1c0a	B	615	648	HOH1291
2	1evv	A	15	48	HOH916
3	1gtr	B	15	48	HOH844
4	1i9v	A	15	48	HOH118
5	1j1u	B	515	549	HOH41
6	1qrs	B	15	48	HOH701
7	1tn1	A	15	48	HOH164
8	1tn2	A	15	48	HOH164
9	3foz	C	15	48	HOH95
10	3v23	A	2505	2610	HOH3784
11	3v2d	A	2505	2610	HOH5398

12	3v2f	A	2505	2610	HOH3836
13	1jj2	0	1377	1683	HOH8555
14	1k73	A	1377	1683	HOH9034
15	1k8a	A	1377	1683	HOH8635
16	1k9m	A	1377	1683	HOH9035
17	1kc8	A	1377	1683	HOH9038
18	1kqs	0	1377	1683	HOH8556
19	1m1k	A	1377	1683	HOH8632
20	1m90	A	1377	1683	HOH6783
21	1nji	A	1377	1683	HOH9036
22	1q81	A	1377	1683	HOH3252
23	1q82	A	1377	1683	HOH8555
24	1q86	A	1377	1683	HOH8557
25	1qvf	0	1377	1683	HOH8554
26	1qvg	0	1377	1683	HOH3243
27	1s72	0	1377	1683	HOH8555
28	1vq5	0	1377	1683	HOH9357
29	1vq5	0	2510	2564	HOH3996
30	1vq7	0	1377	1683	HOH9356
31	1vq8	0	1377	1683	HOH9661
32	1vq9	0	1377	1683	HOH9659
33	1vqm	0	1377	1683	HOH9660
34	1vqn	0	1377	1683	HOH9661
35	1vqo	0	1377	1683	HOH9660
36	1vqp	0	1377	1683	HOH9660
37	1w2b	0	1377	1683	HOH6212
38	1yhq	0	1377	1683	HOH9533
39	1yij	0	2510	2564	HOH3690
40	1yj9	0	1377	1683	HOH8858
41	1yjw	0	1377	1683	HOH7584
42	2otl	0	1377	1683	HOH9036
43	2qex	0	1377	1683	HOH3564
44	3cc2	0	1377	1683	HOH8556
45	3cc4	0	1377	1683	HOH9041
46	3cce	0	1377	1683	HOH9041
47	3ccj	0	1377	1683	HOH9044
48	3ccm	0	1377	1683	HOH3768
49	3ccq	0	1377	1683	HOH9044
50	3cd6	0	1377	1683	HOH9041
51	3cma	0	1377	1683	HOH2956
52	3cme	0	1377	1683	HOH2956
53	3cpw	0	1377	1683	HOH9531
54	3cxc	0	1377	1683	HOH3033
55	3g71	0	1377	1683	HOH3056
56	3i56	0	1377	1683	HOH3051
57	1ehz	A	15	48	HOH104
58	1jj2	0	1856	1873	HOH9721
59	1k73	A	1856	1873	HOH3196
60	1k8a	A	1856	1873	HOH9784
61	1k9m	A	1856	1873	HOH3190
62	1kc8	A	1856	1873	HOH3195

63	1kd1	A	1377	1683	HOH9033
64	1kd1	A	1856	1873	HOH3196
65	1kqs	0	1856	1873	HOH9705
66	1m1k	A	1856	1873	HOH9797
67	1m90	A	1856	1873	HOH9722
68	1n8r	A	1377	1683	HOH9438
69	1n8r	A	1856	1873	HOH3587
70	1nji	A	1856	1873	HOH3193
71	1q81	A	1856	1873	HOH9712
72	1q82	A	1856	1873	HOH9708
73	1q86	A	1856	1873	HOH9720
74	1qvf	0	1856	1873	HOH9713
75	1qvg	0	1856	1873	HOH9703
76	1s72	0	1856	1873	HOH9717
77	1vq4	0	1377	1683	HOH9355
78	1vq4	0	1856	1873	HOH3505
79	1vq5	0	1856	1873	HOH6138
80	1vq6	0	1377	1683	HOH9357
81	1vq6	0	1856	1873	HOH3505
82	1vq7	0	1856	1873	HOH6137
83	1vq8	0	1856	1873	HOH3798
84	1vq9	0	1856	1873	HOH3777
85	1vqk	0	1377	1683	HOH9661
86	1vqk	0	1856	1873	HOH3808
87	1vql	0	1377	1683	HOH9660
88	1vql	0	1856	1873	HOH3798
89	1vqm	0	1856	1873	HOH3803
90	1vqn	0	1856	1873	HOH3801
91	1vqo	0	1856	1873	HOH3819
92	1vqp	0	1856	1873	HOH3810
93	1w2b	0	1856	1873	HOH7128
94	1w2b	0	2510	2564	HOH8275
95	1yhq	0	1856	1873	HOH3699
96	1yi2	0	1377	1683	HOH9033
97	1yij	0	1377	1683	HOH9034
98	1yj9	0	1856	1873	HOH5664
99	1yjn	0	1377	1683	HOH9033
100	2otj	0	1377	1683	HOH9035
101	2otj	0	1856	1873	HOH3214
102	2otl	0	1856	1873	HOH3205
103	3cc2	0	1856	1873	HOH9742
104	3cc7	0	1377	1683	HOH9042
105	3cc7	0	1856	1873	HOH5905
106	3cce	0	1856	1873	HOH3217
107	3ccj	0	1856	1873	HOH5804
108	3ccl	0	1377	1683	HOH-9043
109	3ccl	0	1856	1873	HOH5881
110	3ccm	0	1856	1873	HOH3222
111	3ccq	0	1856	1873	HOH5897
112	3ccr	0	1377	1683	HOH9042
113	3ccr	0	1856	1873	HOH5846

114	3ccs	0	1377	1683	HOH9042
115	3ccs	0	1856	1873	HOH5858
116	3ccu	0	1377	1683	HOH9040
117	3ccu	0	1856	1873	HOH5886
118	3ccv	0	1377	1683	HOH9039
119	3ccv	0	1856	1873	HOH5872
120	3cma	0	1856	1873	HOH4099
121	3cme	0	1856	1873	HOH4107
122	3cpw	0	1856	1873	HOH3648
123	3cxc	0	1856	1873	HOH4163
124	3g6e	0	1377	1683	HOH3056
125	3cme	0	2510	2564	HOH5408
126	3cpw	0	2510	2564	HOH4116
127	3i56	0	2510	2564	HOH8405
128	3g71	0	2510	2564	HOH8380
129	3cc2	0	2510	2564	HOH3235
130	3cc4	0	2510	2564	HOH3722
131	3cc7	0	2510	2564	HOH3715
132	3ccl	0	2510	2564	HOH3705
133	3ccm	0	2510	2564	HOH4553
134	3ccq	0	2510	2564	HOH4554
135	3ccs	0	2510	2564	HOH3681
136	2otj	0	2510	2564	HOH3698
137	2otl	0	2510	2564	HOH3692
138	1yhq	0	2510	2564	HOH4178
139	1yi2	0	2510	2564	HOH3678
140	1yjw	0	2510	2564	HOH9140
141	1jj2	0	2510	2564	HOH3208
142	1k73	A	2510	2564	HOH3676
143	1k8a	A	2510	2564	HOH3270
144	1k9m	A	2510	2564	HOH3672
145	1kc8	A	2510	2564	HOH3686
146	1kd1	A	2510	2564	HOH3677
147	1kqs	0	2510	2564	HOH3189
148	1m1k	A	2510	2564	HOH3275
149	1m90	A	2510	2564	HOH3209
150	1n8r	A	2510	2564	HOH4076
151	1nji	A	2510	2564	HOH3673
152	1q81	A	2510	2564	HOH3194
153	1q82	A	2510	2564	HOH3193
154	1q86	A	2510	2564	HOH3207
155	1qvf	0	2510	2564	HOH3195
156	1s72	0	2510	2564	HOH3203
157	1vq6	0	2510	2564	HOH3989
158	1vq7	0	2510	2564	HOH4005
159	3g6e	0	1377	1683	HOH3056
160	3cpw	0	2510	2564	HOH4116

GC WW trans/wa

	PDB	Ch_ID	G	C	additional moieties
1	1ehz	A	15	48	HOH121

2	1ivs	C	915	947	HOH207
3	1ivs	D	915	947	HOH127
4	1tra	A	15	48	HOH113
5	1jj2	0	1856	1873	HOH9262
6	1k73	A	1856	1873	HOH9736
7	1k8a	A	1856	1873	HOH9328
8	1k9m	A	1856	1873	HOH9727
9	1kc8	A	1856	1873	HOH9734
10	1kd1	A	1377	1683	HOH9959
11	1kd1	A	1856	1873	HOH9729
12	1kqs	0	1856	1873	HOH9250
13	1m1k	A	1856	1873	HOH9333
14	1m90	A	1856	1873	HOH9258
15	1n8r	A	1377	1683	HOH3358
16	1n8r	A	1856	1873	HOH3132
17	1nji	A	1856	1873	HOH9732
18	1q81	A	1856	1873	HOH9251
19	1q82	A	1856	1873	HOH9247
20	1q86	A	1856	1873	HOH9260
21	1qvf	0	1856	1873	HOH9259
22	1qvg	0	1856	1873	HOH9250
23	1s72	0	1856	1873	HOH9260
24	1vq4	0	1377	1683	HOH3283
25	1vq4	0	1856	1873	HOH3054
26	1vq5	0	1856	1873	HOH3060
27	1vq6	0	1377	1683	HOH3280
28	1vq6	0	1856	1873	HOH3055
29	1vq7	0	1856	1873	HOH3058
30	1vq8	0	1856	1873	HOH3355
31	1vq9	0	1856	1873	HOH3330
32	1vqk	0	1377	1683	HOH3585
33	1vqk	0	1856	1873	HOH3356
34	1vql	0	1377	1683	HOH3575
35	1vql	0	1856	1873	HOH3351
36	1vqm	0	1856	1873	HOH3353
37	1vqn	0	1856	1873	HOH3350
38	1vqo	0	1856	1873	HOH3365
39	1vqp	0	1856	1873	HOH3362
40	1w2b	0	1856	1873	HOH7187
41	1w2b	0	2510	2564	HOH8270
42	1yhq	0	1856	1873	HOH3240
43	1yi2	0	1377	1683	HOH9974
44	1yi2	0	1856	1873	HOH9740
45	1yij	0	1377	1683	HOH9975
46	1yij	0	1856	1873	HOH9741
47	1yj9	0	1856	1873	HOH9556
48	1yjn	0	1377	1683	HOH9974
49	1yjn	0	1856	1873	HOH9742
50	1yjw	0	1856	1873	HOH8034
51	2otj	0	1377	1683	HOH9982
52	2otj	0	1856	1873	HOH9748

53	2otl	0	1856	1873	HOH9743
54	2qex	0	1856	1873	HOH9567
55	3cc2	0	1856	1873	HOH9276
56	3cc4	0	1856	1873	HOH9767
57	3cc7	0	1377	1683	HOH3007
58	3cc7	0	1856	1873	HOH9768
59	3cce	0	1856	1873	HOH9760
60	3ccj	0	1856	1873	HOH9752
61	3ccl	0	1377	1683	HOH9990
62	3ccl	0	1856	1873	HOH9753
63	3ccm	0	1856	1873	HOH9763
64	3ccq	0	1856	1873	HOH9756
65	3ccr	0	1377	1683	HOH9976
66	3ccr	0	1856	1873	HOH9750
67	3ccs	0	1377	1683	HOH9975
68	3ccs	0	1856	1873	HOH9745
69	3ccu	0	1377	1683	HOH9983
70	3ccu	0	1856	1873	HOH9751
71	3ccv	0	1377	1683	HOH9981
72	3ccv	0	1856	1873	HOH9748
73	3cd6	0	1856	1873	HOH9760
74	3cma	0	1856	1873	HOH3658
75	3cme	0	1856	1873	HOH3655
76	3cpw	0	1856	1873	HOH3196
77	3cxc	0	1856	1873	HOH3712
78	3g4s	0	1856	1873	HOH5631
79	3g6e	0	1377	1683	HOH6593
80	3g6e	0	1856	1873	HOH5751
81	3g71	0	1856	1873	HOH5840
82	3i56	0	1856	1873	HOH5815

GC WW trans/wcwa

	PDB	Ch_ID	G	C	additional moieties
1	1ehz	A	15	48	HOH121, HOH104
2	1jj2	0	1856	1873	HOH9262; HOH9721
3	1k73	A	1856	1873	HOH9736; HOH3196
4	1k8a	A	1856	1873	HOH9328; HOH9784
5	1k9m	A	1856	1873	HOH9727; HOH3190
6	1kc8	A	1856	1873	HOH9734; HOH3195
7	1kd1	A	1377	1683	HOH9959; HOH9033
8	1kd1	A	1856	1873	HOH9729; HOH3196
9	1kqs	0	1856	1873	HOH9250; HOH9705
10	1m1k	A	1856	1873	HOH9333; HOH9797
11	1m90	A	1856	1873	HOH9258; HOH9722
12	1n8r	A	1377	1683	HOH3358; HOH9438
13	1n8r	A	1856	1873	HOH3132; HOH3587
14	1nji	A	1856	1873	HOH9732; HOH3193
15	1q81	A	1856	1873	HOH9251; HOH9712
16	1q82	A	1856	1873	HOH9247; HOH9708
17	1q86	A	1856	1873	HOH9260; HOH9720
18	1qvf	0	1856	1873	HOH9259; HOH9713

19	1qvg	0	1856	1873	HOH9250; HOH9703
20	1s72	0	1856	1873	HOH9260; HOH9717
21	1vq4	0	1377	1683	HOH3283; HOH9355
22	1vq4	0	1856	1873	HOH3054; HOH3505
23	1vq5	0	1856	1873	HOH3060; HOH6138
24	1vq6	0	1377	1683	HOH3280; HOH9357
25	1vq6	0	1856	1873	HOH3055; HOH3505
26	1vq7	0	1856	1873	HOH3058; HOH6137
27	1vq8	0	1856	1873	HOH3355; HOH3798
28	1vq9	0	1856	1873	HOH3330; HOH3777
29	1vqk	0	1377	1683	HOH3585; HOH9661
30	1vqk	0	1856	1873	HOH3356; HOH3808
31	1vql	0	1377	1683	HOH3575; HOH9660
32	1vql	0	1856	1873	HOH3351; HOH3798
33	1vqm	0	1856	1873	HOH3353; HOH3803
34	1vqn	0	1856	1873	HOH3350; HOH3801
35	1vqo	0	1856	1873	HOH3365; HOH3819
36	1vqp	0	1856	1873	HOH3362; HOH3810
37	1w2b	0	1856	1873	HOH7187; HOH7128
38	1w2b	0	2510	2564	HOH8270; HOH8275
39	1yhq	0	1856	1873	HOH3240; HOH3699
40	1yi2	0	1377	1683	HOH9974; HOH9033
41	1yij	0	1377	1683	HOH9975; HOH9034
42	1yj9	0	1856	1873	HOH9556; HOH5664
43	1yjn	0	1377	1683	HOH9974; HOH9033
44	2otj	0	1377	1683	HOH9982; HOH9035
45	2otj	0	1856	1873	HOH9748; HOH3214
46	2otl	0	1856	1873	HOH9743; HOH3205
47	3cc2	0	1856	1873	HOH9276; HOH9742
48	3cc7	0	1377	1683	H3007; HOH9042
49	3cc7	0	1856	1873	HOH9768; HOH5905
50	3cce	0	1856	1873	HOH9760; HOH3217
51	3ccj	0	1856	1873	HOH9752; HOH5804
52	3ccl	0	1377	1683	HOH9990; HOH-9043
53	3ccl	0	1856	1873	HOH9753; HOH5881
54	3ccm	0	1856	1873	HOH9763; HOH3222
55	3ccq	0	1856	1873	HOH9756; HOH5897
56	3ccr	0	1377	1683	HOH9976; HOH9042
57	3ccr	0	1856	1873	HOH9750; HOH5846
58	3ccs	0	1377	1683	HOH9975; HOH9042
59	3ccs	0	1856	1873	HOH9745; HOH5858
60	3ccu	0	1377	1683	HOH9983; HOH9040
61	3ccu	0	1856	1873	HOH9751; HOh5886
62	3ccv	0	1377	1683	HOH9981; HOH9039
63	3ccv	0	1856	1873	HOH9748; HOH5872
64	3cma	0	1856	1873	HOH3658; HOH4099
65	3cme	0	1856	1873	HOH3655; HOH4107
66	3cpw	0	1856	1873	HOH3196; HOH3648
67	3cxc	0	1856	1873	HOH3712; HOH4163
68	3g6e	0	1377	1683	HOH6593; HOH3056

GC WW trans/Ph					
	PDB	Ch_ID	G	C	additional moieties
1	1j5a	A	1284	1631	A763; OP1
2	1jzx	A	1284	1631	A763; OP1
3	1jzy	A	1284	1631	A763; OP1
4	1k01	A	1284	1631	A763; OP1
5	1vs6	B	1271	1615	A750; OP1
6	1vs8	B	1271	1615	A750; OP1
7	1vt2	A	1271	1615	A750; OP1
8	1y69	0	1284	1631	A763; OP1
9	2aw4	B	1271	1615	A750; OP1
10	2awb	B	1271	1615	A750; OP1
11	2d3o	0	1284	1631	A763; OP1
12	2i2t	B	1271	1615	A750; OP1
13	2i2v	B	1271	1615	A750; OP1
14	2j01	A	1271	1615	A750; OP1
15	2j03	A	1271	1615	A750; OP1
16	2ogm	0	1284	1631	A763; OP1
17	2qam	B	1271	1615	A750; OP1
18	2qao	B	1271	1615	A750; OP1
19	2qbe	B	1271	1615	A750; OP1
20	2qbg	B	1271	1615	A750; OP1
21	2qoz	B	1271	1615	A750; OP1
22	2qp1	B	1271	1615	A750; OP1
23	2wdi	A	1271	1615	A750; OP1
24	2wdj	A	1271	1615	A750; OP1
25	2wdl	A	1271	1615	A750; OP1
26	2wdn	A	1271	1615	A750; OP1
27	2wh2	A	1271	1615	A750; OP1
28	2wh4	A	1271	1615	A750; OP1
29	2x9s	A	1271	1615	A750; OP1
30	2x9u	A	1271	1615	A750; OP1
31	2xg0	A	1271	1615	A750; OP1
32	2xg2	A	1271	1615	A750; OP1
33	2xqe	A	1271	1615	A750; OP1
34	2y0v	A	1271	1615	A750; OP1
35	2y0x	A	1271	1615	A750; OP1
36	2y0z	A	1271	1615	A750; OP1
37	2y11	A	1271	1615	A750; OP1
38	2y13	A	1271	1615	A750; OP1
39	2y15	A	1271	1615	A750; OP1
40	2y17	A	1271	1615	A750; OP1
41	2y19	A	1271	1615	A750; OP1
42	2zjq	X	1284	1631	A763; OP1
43	2zjr	X	1284	1631	A763; OP1
44	3cf5	X	1284	1631	A763; OP1
45	3d5b	A	1271	1615	A750; OP1
46	3d5d	A	1271	1615	A750; OP1
47	3df2	B	1271	1615	A750; OP1
48	3df4	B	1271	1615	A750; OP1

49	3dll	X	1284	1631	A763; OP1
50	3f1f	A	1271	1615	A750; OP1
51	3f1h	A	1271	1615	A750; OP1
52	3hux	A	1271	1615	A750; OP1
53	3huz	A	1271	1615	A750; OP1
54	3i1n	A	1271	1615	A750; OP1
55	3i1p	A	1271	1615	A750; OP1
56	3i8f	A	1271	1615	A750; OP1
57	3i8i	A	1271	1615	A750; OP1
58	3i9c	A	1271	1615	A750; OP1
59	3i9e	A	1271	1615	A750; OP1
60	3kir	A	1271	1615	A750; OP1
61	3kit	A	1271	1615	A750; OP1
62	3kni	A	1271	1615	A750; OP1
63	3knk	A	1271	1615	A750; OP1
64	3knm	A	1271	1615	A750; OP1
65	3kno	A	1271	1615	A750; OP1
66	3oas	A	1271	1615	A750; OP1
67	3oat	A	1271	1615	A750; OP1
68	3ofc	A	1271	1615	A750; OP1
69	3ofd	A	1271	1615	A750; OP1
70	3ofq	A	1271	1615	A750; OP1
71	3ofr	A	1271	1615	A750; OP1
72	3ofz	A	1271	1615	A750; OP1
73	3og0	A	1271	1615	A750; OP1
74	3oh5	A	1271	1615	A750; OP1
75	3oh7	A	1271	1615	A750; OP1
76	3ohj	A	1271	1615	A750; OP1
77	3ohk	A	1271	1615	A750; OP1
78	3ohz	A	1271	1615	A750; OP1
79	3oi1	A	1271	1615	A750; OP1
80	3oi3	A	1271	1615	A750; OP1
81	3oi5	A	1271	1615	A750; OP1
82	3orb	A	1271	1615	A750; OP1
83	3pyo	A	1271	1615	A750; OP1
84	3pyr	A	1271	1615	A750; OP1
85	3pyt	A	1271	1615	A750; OP1
86	3pyv	A	1271	1615	A750; OP1
87	3r8s	A	1271	1615	A750; OP1
88	3r8t	A	1271	1615	A750; OP1
89	3sgf	A	1271	1615	A750; OP1
90	3tve	A	1271	1615	A750; OP1
91	3tvh	A	1271	1615	A750; OP1
92	3uxq	A	1271	1615	A750; OP1
93	3uxr	A	1271	1615	A750; OP1
94	3uye	A	1271	1615	A750; OP1
95	3uyg	A	1271	1615	A750; OP1
96	3uz1	A	1271	1615	A750; OP1
97	3uz2	A	1271	1615	A750; OP1
98	3uz8	A	1271	1615	A750; OP1
99	3uz9	A	1271	1615	A750; OP1

100	3uzf	A	1271	1615	A750; OP1
101	3uzh	A	1318	1662	OP1; A768
102	3uzk	A	1271	1615	A750; OP1
103	3uzn	A	1271	1615	A750; OP1
104	3v23	A	1271	1615	A750; OP1
105	3v25	A	1271	1615	A750; OP1
106	3v27	A	1271	1615	A750; OP1
107	3v29	A	1271	1615	A750; OP1
108	3v2d	A	1271	1615	A750; OP1
109	3v2f	A	1271	1615	A750; OP1
110	4abs	A	1271	1615	A750; OP1
111	4dha	A	1271	1615	A750; OP1
112	4dhc	A	1271	1615	A750; OP1
113	4gar	A	1271	1615	A750; OP1
114	4gau	A	1271	1615	A750; OP1
115	1ffk	0	2510	2564	C2508; OP1
116	1fg0	A	2510	2564	C2508; OP1
117	1j5a	A	2454	2508	U2452;OP1
118	1vq4	0	2510	2564	C2508; OP1
119	1vq8	0	2510	2564	C2508; OP1
120	1vqk	0	2510	2564	C2508; OP1
121	1vql	0	2510	2564	C2508; OP1
122	1vqm	0	2510	2564	C2508; OP1
123	1vqn	0	2510	2564	C2508; OP1
124	1vqo	0	2510	2564	C2508; OP1
125	1vqp	0	2510	2564	C2508; OP1
126	1vs6	B	2475	2529	U2573; OP1
127	1vs8	B	2475	2529	U2573; OP1
128	1vt2	A	2475	2529	U2473; OP1
129	1yit	0	2510	2564	C2508; OP1
130	1yj9	0	2510	2564	C2508; OP1
131	1yjn	0	2510	2564	C2508; OP1
132	2aw4	B	2475	2529	U2473; OP1
133	2awb	B	2475	2529	U2473; OP1
134	2i2t	B	2475	2529	U2473; OP1
135	2i2v	B	2475	2529	U2473; OP1
136	2j01	A	2475	2529	U2473; OP1
137	2j03	A	2475	2529	U2473; OP1
138	2qa4	0	2510	2564	C2508; OP1
139	2qam	B	2475	2529	U2473; OP1
140	2qao	B	2475	2529	U2473; OP1
141	2qbe	B	2475	2529	U2473; OP1
142	2qbg	B	2475	2529	U2473; OP1
143	2qex	0	2510	2564	C2505; OP1
144	2qoz	B	2475	2529	U2473; OP1
145	2qp1	B	2475	2529	U2473; OP1
146	2v47	A	2475	2529	U2473; OP1
147	2v49	A	2475	2529	U2473; OP1
148	2wdi	A	2475	2529	U2473; OP1
149	2wdj	A	2475	2529	U2473; OP1
150	2wdl	A	2475	2529	U2473; OP1

151	2wdn	A	2475	2529	U2473; OP1
152	2wh2	A	2475	2529	U2473; OP1
153	2wh4	A	2475	2529	U2473; OP1
154	2x9s	A	2475	2529	U2473; OP1
155	2x9u	A	2475	2529	U2473; OP1
156	2xg0	A	2475	2529	U2473; OP1
157	2xqe	A	2475	2529	U2473; OP1
158	2y0v	A	2475	2529	U2473; OP1
159	2y0x	A	2475	2529	U2473; OP1
160	2y0z	A	2475	2529	U2473; OP1
161	2y11	A	2475	2529	U2473; OP1
162	2y13	A	2475	2529	U2473; OP1
163	2y15	A	2475	2529	U2473; OP1
164	2y17	A	2475	2529	U2473; OP1
165	2y19	A	2475	2529	U2473; OP1
166	3g6e	0	2510	2564	C2508; OP1
167	3ccu	0	2510	2564	C2508; OP1
168	3ccv	0	2510	2564	C2508; OP1
169	3cma	0	2510	2564	C2508; OP1
170	3cme	0	2510	2564	C2508; OP1
171	3cpw	0	2510	2564	C2508; OP1
172	3i56	0	2510	2564	C2508 OP1
173	3g71	0	2510	2564	C2508 OP1
174	3cc2	0	2510	2564	C2508; OP1
175	3cc4	0	2510	2564	C2508; OP1
176	3cc7	0	2510	2564	C2508; OP1
177	3ccl	0	2510	2564	C2508; OP1
178	3ccm	0	2510	2564	C2508; OP1
179	3ccq	0	2510	2564	C2508; OP1
180	3ccs	0	2510	2564	C2508; OP1
181	2otj	0	2510	2564	C2508; OP1
182	2otl	0	2510	2564	C2508; OP1
183	1yhq	0	2510	2564	C2508; OP1
184	1yi2	0	2510	2564	C2508; OP1
185	1yjw	0	2510	2564	C2508; OP1
186	1jj2	0	2510	2564	C2508; OP1
187	1k73	A	2510	2564	C2508; OP1
188	1k8a	A	2510	2564	C2508; OP1
189	1k9m	A	2510	2564	C2508; OP1
190	1kc8	A	2510	2564	C2508; OP1
191	1kd1	A	2510	2564	C2508; OP1
192	1kqs	0	2510	2564	C2508; OP1
193	1m1k	A	2510	2564	C2508; OP1
194	1m90	A	2510	2564	C2508; OP1
195	1n8r	A	2510	2564	C2508; OP1
196	1nji	A	2510	2564	C2508; OP1
197	1q81	A	2510	2564	C2508; OP1
198	1q82	A	2510	2564	C2508; OP1
199	1q86	A	2510	2564	C2508; OP1
200	1qvf	0	2510	2564	C2508; OP1
201	1qvg	0	2510	2564	C2508; OP1

202	1s72	0	2510	2564	C2508; OP1
203	1vq6	0	2510	2564	C2508; OP1
204	1vq7	0	2510	2564	C2508; OP1
205	3cce	0	2510	2564	C2508; OP1
206	3cxc	0	2510	2564	C2508; OP1
207	3d5b	A	2475	2529	U2473; OP1
208	3d5d	A	2475	2529	U2473; OP1
209	3df2	B	2475	2529	U2473; OP1
210	3df4	B	2475	2529	U2473; OP1
211	3f1f	A	2475	2529	U2473; OP1
212	3f1h	A	2475	2529	U2473; OP1
213	3hux	A	2475	2529	U2473; OP1
214	3huz	A	2475	2529	U2473; OP1
215	3i1n	A	2475	2529	U2473; OP1
216	3i1p	A	2475	2529	U2473; OP1
217	3i8i	A	2475	2529	U2473; OP1
218	3i9e	A	2475	2529	U2473; OP1
219	3kir	A	2475	2529	U2473; OP1
220	3kit	A	2475	2529	U2473; OP1
221	3kni	A	2475	2529	U2473; OP1
222	3knk	A	2475	2529	U2473; OP1
223	3knm	A	2475	2529	U2473; OP1
224	3kno	A	2475	2529	U2473; OP1
225	3oas	A	2475	2529	U2473; OP1
226	3oat	A	2475	2529	U2473; OP1
227	3ofc	A	2475	2529	U2473; OP1
228	3ofd	A	2475	2529	U2473; OP1
229	3ofq	A	2475	2529	U2473; OP1
230	3ofr	A	2475	2529	U2473; OP1
231	3ofz	A	2475	2529	U2473; OP1
232	3og0	A	2475	2529	U2473; OP1
233	3oh5	A	2475	2529	U2473; OP1
234	3oh7	A	2475	2529	U2473; OP1
235	3ohj	A	2475	2529	U2473; OP1
236	3ohk	A	2475	2529	U2473; OP1
237	3ohz	A	2475	2529	U2473; OP1
238	3oi1	A	2475	2529	U2473; OP1
239	3oi3	A	2475	2529	U2473; OP1
240	3oi5	A	2475	2529	U2473; OP1
241	3orb	A	2475	2529	U2473; OP1
242	3ow2	0	2510	2564	C2508; OP1
243	3pyo	A	2475	2529	U2473; OP1
244	3pyr	A	2475	2529	U2473; OP1
245	3pyt	A	2475	2529	U2473; OP1
246	3pyv	A	2475	2529	U2473; OP1
247	3r8s	A	2475	2529	U2473; OP1
248	3r8t	A	2475	2529	U2473; OP1
249	3sgf	A	2475	2529	U2473; OP1
250	3tve	A	2475	2529	U2473; OP1
251	3u5d	0	2844	2898	U2842; OP1
252	3u5h	0	2844	2898	U2842; OP1

253	3uxq	A	2475	2529	U2473; OP1
254	3uxr	A	2475	2529	U2473; OP1
255	3uye	A	2475	2529	U2473; OP1
256	3uz1	A	2475	2529	U2473; OP1
257	3uz9	A	2475	2529	U2473; OP1
258	3v2d	A	2475	2529	U2473; OP1
259	3v2f	A	2475	2529	U2473; OP1
260	4abs	A	2475	2529	U2473; OP1
261	4dha	A	2475	2529	U2473; OP1
262	4dhc	A	2475	2529	U2473; OP1
263	4gar	A	2475	2529	U2473; OP1
264	4gau	A	2475	2529	U2473; OP1
265	3cce	0	2510	2564	C2508; OP1
266	3cxc	0	2510	2564	C2508; OP1
267	3d5b	A	2475	2529	U2473; OP1
268	3cc7	0	2510	2564	C2508; OP1
269	3ccl	0	2510	2564	C2508; OP1
270	3ccm	0	2510	2564	C2508; OP1

GC WW trans/Ph-wc

	PDB	Ch_ID	G	C	additional moieties
1	3g6e	0	2510	2564	C2508; OP1; HOH8361
2	3ccu	0	2510	2564	C2508; OP1; HOH3695
3	3ccv	0	2510	2564	C2508; OP1; HOH3690
4	3cma	0	2510	2564	C2508; OP1; HOH5399
5	3cme	0	2510	2564	C2508; OP1; HOH5408
6	3cc4	0	2510	2564	C2508; OP1; HOH3722
7	3cc7	0	2510	2564	C2508; OP1; HOH3715
8	3ccl	0	2510	2564	C2508; OP1; HOH3705
9	3ccm	0	2510	2564	C2508; OP1; HOH4553
10	3ccq	0	2510	2564	C2508; OP1; HOH4554
11	3ccs	0	2510	2564	C2508; OP1; HOH3681
12	2otj	0	2510	2564	C2508; OP1; HOH3698
13	2otl	0	2510	2564	C2508; OP1; HOH3692
14	1yhq	0	2510	2564	C2508; OP1;HOH4178
15	1yi2	0	2510	2564	C2508; OP1; HOH3678
16	1yjw	0	2510	2564	C2508; OP1; HOH9140
17	1jj2	0	2510	2564	C2508; OP1;HOH3208
18	1k73	A	2510	2564	C2508; OP1; HOH3676
19	1k8a	A	2510	2564	C2508; OP1; HOH3270
20	1k9m	A	2510	2564	C2508; OP1; HOH3672
21	1kc8	A	2510	2564	C2508; OP1; HOH3686
22	1kqs	0	2510	2564	C2508; OP1; HOH3189
23	1m1k	A	2510	2564	C2508; OP1; HOH3275
24	1m90	A	2510	2564	C2508; OP1; HOH3209
25	1n8r	A	2510	2564	C2508; OP1; HOH4076
26	1nji	A	2510	2564	C2508; OP1; HOH3673
27	1q81	A	2510	2564	C2508; OP1; HOH3194
28	1q82	A	2510	2564	C2508; OP1; HOH3193
29	1q86	A	2510	2564	C2508; OP1;HOH3207
30	1qvf	0	2510	2564	C2508; OP1; HOH3195

31	1qvg	0	2510	2564	C2508; OP1; HOH4000
32	1s72	0	2510	2564	C2508; OP1; HOH3203
33	1vq6	0	2510	2564	C2508; OP1; HOH3989
34	1vq7	0	2510	2564	C2508; OP1; HOH4005

Table S2. Gas phase energy values of $E^{complex}$, E^{GC} pair and $E^{surrounding}$. All energy values are reported in hartrees.

Motifs	Ecomplex	EGC	Esurr
GCC tWW/tSW	-1447.723788	-1014.253797	-433.430338
GCU tWW/Intermediate-O4(U)	-1543.933925	-1014.425531	-529.640718
GCH2U(rG) tWW/tSW	-1968.575016	-1014.256121	-954.285602
GrCrG tWW/cSS	-2435.623967	-1434.516661	-1001.054256
GGCU(i) tHS/tWW/tWW	-2048.353308	-1014.256659	-1034.063046
GGCU(ii) tHS/tWW/tWW	-2048.398868	-1014.262053	-1034.104641
GGCU(iii) tHS/tWW/tWW	-2048.291134	-1014.258649	-1033.995100
GC tWW/wc	-1090.596221	-1014.258186	-76.322021
GC tWW/wa	-1090.597034	-1014.255676	-76.321671
GC tWW/wcwa	-1166.935024	-1014.257603	-152.642978
GC tWW/OP1	-1735.494426	-1014.248827	-721.185484
GC tWW/OP1wc	-1811.840961	-1014.251779	797.511614

Gas phase and in water optimized geometries for the studied motifs:

```
51
1H3E_triplet_gas E =  -1450.94343505 A.U.
  C     -5.301566    -0.473798     0.000801
  N     -0.628648    -2.432827     0.016992
  C     -1.002094    -1.118383     0.034005
  N     -0.028467    -0.201109     0.062767
  N     -2.278404    -0.733878     0.026091
  C     -3.142604    -1.775519     0.001413
  C     -2.865784    -3.136454    -0.017160
  C     -1.492558    -3.563167    -0.010363
  O     -1.027412    -4.685109    -0.024184
  N     -4.032116    -3.872302    -0.039918
  C     -4.982492    -2.985963    -0.035273
  N     -4.511750    -1.682380    -0.010328
  C      1.036960     6.023082    -0.024175
  N     -0.045953     5.046215    -0.011443
  C      0.305038     3.671829     0.000452
  O      1.502703     3.391305    -0.000294
  N     -0.693575     2.760209     0.012037
  C     -1.968914     3.130545     0.011761
  N     -2.905333     2.174225     0.022703
  C     -2.347702     4.512731     0.000386
  C     -1.347439     5.421814    -0.010898
  C      4.741520    -3.815742     0.005807
  N      4.544627    -2.369101    -0.000896
  C      3.210834    -1.891716     0.016708
  O      2.305096    -2.719978     0.036957
  N      3.007895    -0.551278     0.010373
  C      4.025277     0.298121    -0.011487
  N      3.761822     1.611756    -0.016787
  C      5.383446    -0.160224    -0.029137
  C      5.580448    -1.498706    -0.022925
  H     -5.099368     0.114375     0.897415
  H      0.954358    -0.450510     0.047643
  H     -6.041332    -3.191329    -0.048577
  H      1.665566     5.899424     0.855572
  H     -2.662622     1.171843     0.026453
  H     -3.380503     4.823136     0.000393
  H     -1.536310     6.486077    -0.020172
  H      4.294425    -4.250785     0.897307
  H      2.814118     1.980551    -0.006424
  H      6.217291     0.523460    -0.046896
  H      6.569520    -1.934741    -0.035272
  H      5.808373    -4.025420    -0.014300
  H      4.258595    -4.263320    -0.860347
  H     -5.092521     0.135938    -0.879765
  H     -6.355490    -0.743169    -0.006545
  H      0.608884     7.022609    -0.032249
  H      1.659777     5.882733    -0.905539
  H      0.370814    -2.643920     0.024402
  H      4.515024     2.273274    -0.034006
```

```
  H     -3.873419     2.436050     0.023260
  H     -0.263187     0.786577     0.057205


51
1H3E_cosmo
  C      4.981399    -1.922351    -0.096535
  N     -0.068938    -2.412864     0.046830
  C      0.682890    -1.264230     0.074962
  N      0.020500    -0.106367     0.155522
  N      2.013842    -1.276435     0.035690
  C      2.530027    -2.520102    -0.040893
  C      1.853415    -3.735107    -0.077569
  C      0.426279    -3.725822    -0.030616
  O     -0.350335    -4.680069    -0.050717
  N      2.745651    -4.787921    -0.161364
  C      3.920449    -4.219352    -0.175098
  N      3.859747    -2.842600    -0.105085
  C      0.501678     6.048295    -0.161251
  N      1.339360     4.857042    -0.039296
  C      0.712864     3.597422    -0.082103
  O     -0.515600     3.559540    -0.218137
  N      1.483594     2.491295     0.027512
  C      2.804824     2.584259     0.176474
  N      3.504330     1.447255     0.275918
  C      3.463017     3.849909     0.229766
  C      2.684287     4.950209     0.116154
  C     -5.551777    -2.496231     0.197912
  N     -4.965711    -1.162055     0.084746
  C     -3.566917    -1.047874     0.090417
  O     -2.898722    -2.088015     0.189684
  N     -3.009590     0.179949    -0.010298
  C     -3.773211     1.269434    -0.117417
  N     -3.178988     2.462129    -0.214866
  C     -5.199696     1.180134    -0.128865
  C     -5.741998    -0.053306    -0.024310
  H      4.891499    -1.205340    -0.911541
  H     -0.996042    -0.066665     0.107107
  H      4.870412    -4.729569    -0.235257
  H     -0.032106     6.036700    -1.109405
  H      3.035905     0.543909     0.218032
  H      4.532589     3.931195     0.351388
  H      3.093840     5.951597     0.143017
  H     -5.221796    -3.123286    -0.627858
  H     -2.169455     2.575487    -0.202081
  H     -5.821589     2.058331    -0.215490
  H     -6.811802    -0.216330    -0.022497
  H     -6.634066    -2.402716     0.177299
  H     -5.241545    -2.964920     1.129585
  H      5.031021    -1.384331     0.850021
  H      5.899301    -2.490020    -0.226432
  H      1.137797     6.927693    -0.109780
  H     -0.230134     6.077090     0.643554
```

H	-1.092214	-2.315389	0.090862
H	-3.741392	3.295033	-0.291954
H	4.504411	1.481145	0.395388
H	0.540880	0.771022	0.106051

53

1N78_triplet_gas E = -1547.30682922 A.U.

N	-1.533327	2.210013	-0.127433
C	-0.469445	1.616887	0.483245
N	-0.638450	0.367585	0.961035
N	0.696061	2.229168	0.646437
C	0.719443	3.488090	0.145757
C	-0.302248	4.180427	-0.489008
C	-1.572363	3.530207	-0.671838
O	-2.589012	3.937706	-1.194174
N	0.123290	5.439684	-0.855288
C	1.359642	5.499098	-0.452939
N	1.790691	4.338822	0.166497
N	4.412736	-2.961518	-0.924637
C	4.272596	-1.802956	-0.146734
O	5.176577	-1.004384	-0.037003
N	3.043537	-1.663290	0.465630
C	1.966733	-2.529505	0.375441
O	0.916942	-2.283761	0.973111
C	2.195251	-3.691928	-0.451104
C	3.389530	-3.852438	-1.055840
N	-5.120173	-1.536926	-0.263390
C	-3.886971	-0.856994	-0.108362
O	-3.869727	0.357906	-0.275036
N	-2.792763	-1.588093	0.221957
C	-2.866149	-2.896718	0.407588
N	-1.740288	-3.555527	0.730441
C	-4.102506	-3.604660	0.267031
C	-5.191588	-2.873742	-0.069598
O	2.603643	0.703379	1.905595
H	-1.420704	-0.205849	0.654927
H	2.021714	6.342444	-0.569167
H	1.400841	-4.407268	-0.579994
H	3.598332	-4.705962	-1.685430
H	-1.777729	-4.535178	0.940795
H	-4.173682	-4.670426	0.415220
H	-6.166382	-3.322356	-0.200147
H	1.947471	1.315254	1.471904
H	2.360761	0.669230	2.834642
H	-2.396568	1.671622	-0.219565
H	0.185702	-0.149849	1.224247
H	-0.853446	-3.066590	0.839310
H	2.915666	-0.801889	1.028388
C	3.098820	4.076140	0.732843
H	3.038640	3.951445	1.814015
H	3.529944	3.170933	0.308104
H	3.749747	4.918407	0.509880
C	-6.288798	-0.737617	-0.622032

```
  H     -6.479846     0.011385     0.143797
  H     -6.110509    -0.217137    -1.560490
  H     -7.148384    -1.396250    -0.719610
  C      5.697851    -3.149278    -1.593689
  H      5.889281    -2.328367    -2.282079
  H      6.502607    -3.171861    -0.861883
  H      5.671176    -4.088009    -2.140121


53
1N78_triplet_cosmo E =  -1547.35243037 A.U.
  N     -2.441389     1.295408    -0.055930
  C     -1.139485     1.302561     0.371177
  N     -0.612568     0.125937     0.732944
  N     -0.418623     2.415816     0.453093
  C     -1.094340     3.523716     0.080991
  C     -2.408052     3.615053    -0.363392
  C     -3.186604     2.420153    -0.457753
  O     -4.351538     2.288012    -0.826439
  N     -2.731832     4.927542    -0.650726
  C     -1.643490     5.597863    -0.384346
  N     -0.608940     4.802360     0.063318
  N      5.620129    -1.112789    -0.642194
  C      4.950586     0.019507    -0.189118
  O      5.466893     1.123875    -0.208318
  N      3.674056    -0.204461     0.277170
  C      3.015261    -1.421802     0.336247
  O      1.863927    -1.478516     0.784421
  C      3.775021    -2.541341    -0.151346
  C      5.027202    -2.342003    -0.613114
  N     -3.969807    -3.523167    -0.202631
  C     -3.181424    -2.372378    -0.053255
  O     -3.730111    -1.265999    -0.164693
  N     -1.862601    -2.516186     0.207166
  C     -1.312647    -3.725617     0.324515
  N     -0.003672    -3.806698     0.586800
  C     -2.093639    -4.912649     0.179150
  C     -3.410767    -4.755021    -0.083241
  O      2.268819     2.078010     1.228161
  H     -1.088875    -0.755746     0.531362
  H     -1.515688     6.665024    -0.489877
  H      3.334824    -3.525761    -0.142876
  H      5.631711    -3.157479    -0.988557
  H      0.434738    -4.709576     0.680834
  H     -1.656031    -5.895180     0.271986
  H     -4.078795    -5.596861    -0.210300
  H      1.331162     2.245432     0.970442
  H      2.291861     2.164314     2.190408
  H     -2.927394     0.389110    -0.098431
  H      0.378734     0.058985     0.912754
  H      0.578528    -2.977668     0.670433
  H      3.164364     0.627787     0.614586
  C      0.716030     5.240451     0.469102
```

```
  H      1.480253     4.656881    -0.039806
  H      0.830260     6.288799     0.205421
  H      0.847349     5.126496     1.544780
  C     -5.394303    -3.354032    -0.484748
  H     -5.871742    -2.794874     0.317300
  H     -5.530322    -2.805019    -1.414252
  H     -5.850633    -4.336417    -0.568951
  C      6.980489    -0.928415    -1.149936
  H      6.975859    -0.245367    -1.996833
  H      7.618582    -0.513691    -0.372374
  H      7.365035    -1.894858    -1.461774


86
3uz2_gas_triplet E =  -2440.91313137 A.U.
  C     -7.008602    -2.801101     1.800841
  C     -6.103967    -1.695353     1.304154
  O     -4.763033    -1.898719     1.808783
  C     -5.904730    -1.636670    -0.217326
  O     -6.982909    -1.203063    -1.010162
  C     -4.571005    -0.882192    -0.330401
  O     -4.782169     0.496347    -0.112842
  C     -3.796714    -1.499542     0.856384
  N      0.532403    -3.098030    -1.279431
  C      0.105356    -1.818407    -1.079410
  N      0.905704    -0.835672    -1.545244
  N     -1.047704    -1.530173    -0.493036
  C     -1.753286    -2.637722    -0.125412
  C     -1.400202    -3.971658    -0.289025
  C     -0.150324    -4.298128    -0.916272
  O      0.357401    -5.373047    -1.151826
  N     -2.372615    -4.800580     0.220748
  C     -3.290106    -3.997523     0.674336
  N     -2.979042    -2.658316     0.492258
  C     -2.960512     5.377990    -0.890584
  C     -2.489445     3.942479    -0.927999
  O     -1.244884     3.839928    -1.669314
  C     -2.163679     3.309424     0.423411
  O     -3.348844     2.921129     1.056519
  C     -1.201016     2.170766     0.014965
  O     -1.966541     1.126935    -0.532945
  C     -0.392097     2.888794    -1.094455
  N      0.833668     3.591267    -0.570935
  C      1.932068     2.806569    -0.170620
  O      1.843217     1.577928    -0.236794
  N      3.048220     3.433795     0.278458
  C      3.123369     4.755436     0.289379
  N      4.266115     5.312400     0.745446
  C      2.047589     5.576144    -0.148949
  C      0.928448     4.940542    -0.574411
  N      4.290446    -0.249390    -0.027602
  C      5.148899     0.141174     0.966486
  N      4.808202     1.260074     1.658664
```

N	6.235169	-0.521548	1.288111
C	6.414934	-1.631891	0.544089
C	5.621274	-2.117821	-0.491941
C	4.439612	-1.399493	-0.836895
O	3.598892	-1.659903	-1.696199
N	6.152085	-3.280986	-1.006628
C	7.227551	-3.486989	-0.303342
N	7.449206	-2.518702	0.660802
H	-7.032210	-2.822797	2.890242
H	-8.024325	-2.638090	1.438201
H	-6.456848	-0.729074	1.672010
H	-5.717610	-2.656481	-0.563011
H	-4.066794	-1.067987	-1.280689
H	-3.926775	0.950717	-0.180707
H	-3.129093	-0.757004	1.294808
H	1.878443	-1.076279	-1.742506
H	-4.195101	-4.290984	1.173547
H	-3.913121	5.427293	-0.362948
H	-3.108504	5.763186	-1.898953
H	-3.233134	3.319410	-1.428315
H	-1.615486	4.029450	1.048290
H	-0.555936	1.826265	0.822172
H	-1.533697	0.234048	-0.477829
H	-0.048409	2.184513	-1.848601
H	5.055647	4.716003	0.920368
H	2.112709	6.652370	-0.160749
H	0.066216	5.461759	-0.956069
H	5.506772	1.581620	2.305212
H	7.906080	-4.316279	-0.425697
H	-6.673064	-3.774828	1.442197
H	-2.248439	6.021990	-0.371091
C	-3.193393	2.503247	2.403155
H	-4.186440	2.263439	2.773043
H	-2.563092	1.613088	2.482412
H	-2.759707	3.302099	3.015159
C	-7.680720	-0.013289	-0.652009
H	-8.413795	0.140701	-1.441773
H	-8.216937	-0.126657	0.295527
H	-7.018921	0.847635	-0.591206
H	3.450274	0.308081	-0.176898
H	4.198578	1.953535	1.237156
H	4.423691	6.299671	0.665233
H	0.800854	0.072139	-1.120758
H	1.446870	-3.240059	-1.690190
C	8.539173	-2.450126	1.615502
H	9.499548	-2.415500	1.101514
H	8.416232	-1.543584	2.201608
H	8.522642	-3.310598	2.284339

86

3uz2_cosmo_triplet E = -2440.99600474 A.U.

C	-7.059409	-2.634960	2.099542

C	-6.124436	-1.584454	1.543951
O	-4.755275	-1.929260	1.883613
C	-6.084717	-1.473905	0.013230
O	-7.184678	-0.871941	-0.639136
C	-4.702991	-0.850986	-0.240538
O	-4.756239	0.545106	-0.028544
C	-3.862159	-1.542401	0.856394
N	0.398883	-3.302513	-1.284330
C	0.082273	-2.009614	-0.947655
N	0.990918	-1.069258	-1.235557
N	-1.079122	-1.684555	-0.392087
C	-1.891350	-2.746154	-0.183970
C	-1.647634	-4.080911	-0.491258
C	-0.409148	-4.436479	-1.097772
O	-0.006217	-5.546236	-1.445230
N	-2.717972	-4.871584	-0.122669
C	-3.582356	-4.037202	0.388743
N	-3.140065	-2.728887	0.383496
C	-2.722920	5.342850	-1.569334
C	-2.322047	3.899525	-1.369698
O	-1.004420	3.659659	-1.938115
C	-2.177296	3.434120	0.078778
O	-3.438720	3.168501	0.636671
C	-1.235463	2.223188	-0.068686
O	-1.991647	1.130781	-0.568980
C	-0.268646	2.754407	-1.149913
N	0.922966	3.447442	-0.570057
C	1.976409	2.660616	-0.087602
O	1.885996	1.420508	-0.158923
N	3.058098	3.266541	0.442819
C	3.143920	4.596928	0.489696
N	4.240538	5.138435	1.028994
C	2.102734	5.424654	-0.020891
C	1.019692	4.802937	-0.539141
N	4.420899	-0.210269	0.038943
C	5.269591	0.182031	1.047426
N	4.909908	1.274686	1.750409
N	6.371820	-0.476547	1.358537
C	6.578542	-1.562227	0.591002
C	5.793140	-2.033121	-0.457340
C	4.607851	-1.324388	-0.788662
O	3.786347	-1.591499	-1.679560
N	6.347702	-3.176338	-1.003537
C	7.431025	-3.376949	-0.298572
N	7.627510	-2.433188	0.684094
H	-6.964032	-2.701065	3.183494
H	-8.091316	-2.369463	1.866498
H	-6.344056	-0.612893	1.990494
H	-6.052362	-2.488947	-0.389139
H	-4.320902	-1.085966	-1.235649
H	-3.865164	0.917145	-0.167128
H	-3.110095	-0.858238	1.247394
H	1.949015	-1.324111	-1.472680

```
  H      -4.541935    -4.299756     0.798171
  H      -3.728547     5.493913    -1.175083
  H      -2.734730     5.595223    -2.629641
  H      -3.033062     3.243762    -1.878012
  H      -1.664006     4.205864     0.663918
  H      -0.714515     1.957770     0.848679
  H      -1.534661     0.276041    -0.429818
  H       0.115134     1.946296    -1.766420
  H       4.981704     4.548298     1.368269
  H       2.174875     6.500389    -0.003311
  H       0.191959     5.344071    -0.964880
  H       5.601644     1.631320     2.389422
  H       8.131230    -4.185675    -0.434884
  H      -6.846585    -3.613047     1.666206
  H      -2.043755     6.021578    -1.052350
  C      -3.423225     3.024486     2.055474
  H      -4.454667     2.886153     2.370004
  H      -2.834875     2.156775     2.363519
  H      -3.014710     3.920811     2.530640
  C      -7.798258     0.279314    -0.050042
  H      -8.549460     0.608172    -0.765551
  H      -8.297486     0.030827     0.889132
  H      -7.084021     1.082184     0.118163
  H       3.567866     0.336967    -0.107494
  H       4.263302     1.953077     1.343394
  H       4.355551     6.135783     1.079969
  H       0.893256    -0.154694    -0.814800
  H       1.301789    -3.482082    -1.704336
  C       8.728373    -2.370937     1.633072
  H       9.284013    -1.443440     1.505866
  H       8.350010    -2.424085     2.652397
  H       9.388853    -3.213856     1.450374


76
1SER_gas_quartet E =  -1959.53452383 A.U.
  N       3.697903     2.170218    -0.170597
  C       3.326998     0.938462    -0.629959
  N       2.047126     0.791344    -1.022554
  N       4.168159    -0.082570    -0.727104
  C       5.438694     0.251232    -0.384484
  C       5.918942     1.469210     0.085520
  C       5.002782     2.564605     0.242615
  O       5.193288     3.695558     0.644855
  N       7.277719     1.403626     0.307032
  C       7.603797     0.188014    -0.025347
  N       6.531408    -0.569780    -0.456994
  N       0.765639    -3.991278    -0.184544
  C       1.350043    -2.757557    -0.415406
  O       0.821378    -1.847673    -1.056394
  N       2.626913    -2.624808     0.112766
  C       3.242434    -3.501530     1.006302
  O       4.455094    -3.485151     1.186408
```

C	2.333407	-4.404209	1.651455
C	1.492817	-5.035996	0.574804
C	-5.171489	-1.549525	-0.204038
O	-5.140766	-0.217865	-0.710516
C	-6.537472	-1.713123	0.427353
O	-6.303603	-1.362614	1.784973
C	-7.389166	-0.652538	-0.295590
O	-8.506280	-0.255989	0.529636
C	-6.391661	0.450658	-0.450466
N	-0.957276	4.555167	0.504950
C	-0.026256	3.563651	0.102004
O	1.164138	3.852345	0.097697
N	-0.510061	2.347474	-0.260351
C	-1.805285	2.087623	-0.239490
N	-2.205909	0.852759	-0.601137
C	-2.765211	3.069358	0.162068
C	-2.281135	4.282648	0.522467
H	1.341486	1.461799	-0.746506
H	8.596281	-0.232255	0.014966
H	3.215240	-1.880669	-0.293287
H	1.708973	-3.867981	2.358446
H	2.170717	-5.498027	-0.111799
H	0.750738	-5.625216	1.072328
H	-4.429283	-1.763696	0.536347
H	-7.009597	-2.671262	0.364329
H	-7.828563	-0.971175	-1.217713
H	-8.946046	0.498880	0.131665
H	-6.548404	1.035937	0.431460
H	-3.181824	0.614593	-0.699349
H	-3.822512	2.862172	0.186381
H	-2.928758	5.087255	0.841644
C	-0.591302	-4.236136	-0.640504
H	-1.275247	-3.557060	-0.162702
H	-0.661220	-4.109024	-1.702631
H	-0.888938	-5.245236	-0.394868
C	6.566506	-1.936989	-0.938598
H	6.010415	-2.595440	-0.274220
H	6.140888	-1.992949	-1.940867
H	7.604483	-2.260691	-0.980540
C	-0.423141	5.858320	0.892322
H	0.271751	5.744675	1.721621
H	0.120603	6.301473	0.060583
H	-1.247767	6.504340	1.183524
C	-4.892838	-2.511057	-1.374222
H	-4.058707	-2.151950	-1.940063
H	-4.670809	-3.484912	-0.990526
H	-5.755383	-2.564419	-2.005152
C	-5.001374	-0.785304	1.910701
H	-4.817962	-0.538754	2.935611
H	-4.266309	-1.486938	1.575585
H	-4.945443	0.101743	1.314937
H	1.717507	-0.148993	-1.197073
H	-1.504914	0.202124	-0.916435

```
  H      2.979586     2.887133    -0.096939
  H      2.888134    -5.154394     2.195234
  C     -6.470264     1.441922    -1.626391
  H     -7.267315     2.135130    -1.455861
  H     -5.545574     1.974316    -1.706511
  H     -6.652512     0.905075    -2.533850


76
1SER_cosmo_quartet E =  -1973.12160677 A.U.
  N      3.280569     2.182340    -0.095185
  C      2.997566     0.849048    -0.254384
  N      1.717206     0.475774    -0.132300
  N      3.934495    -0.060706    -0.505937
  C      5.173764     0.463098    -0.606360
  C      5.554900     1.793296    -0.461207
  C      4.553062     2.765882    -0.177065
  O      4.689294     3.982073    -0.014157
  N      6.918800     1.930751    -0.644297
  C      7.335099     0.716343    -0.893986
  N      6.325735    -0.219690    -0.885355
  N      1.347959    -4.467332     0.139768
  C      1.796736    -3.225312    -0.123667
  O      1.161046    -2.371854    -0.751343
  N      3.071483    -2.902452     0.359169
  C      3.803997    -3.615883     1.271899
  O      4.878415    -3.205677     1.686061
  C      3.161727    -4.896164     1.736430
  C      2.253117    -5.487595     0.671301
  C     -5.673703    -0.117933    -1.380333
  O     -5.116478     0.300764    -0.107685
  C     -7.003913    -0.843230    -1.061410
  O     -7.076601    -2.178055    -1.550163
  C     -7.142297    -0.664046     0.468332
  O     -7.855062    -1.732447     1.067336
  C     -5.698720    -0.480921     0.950400
  N     -0.959797     4.803265     0.632275
  C     -0.044381     3.764366     0.426000
  O      1.169608     4.034220     0.448924
  N     -0.509508     2.513813     0.211093
  C     -1.821859     2.266189     0.193435
  N     -2.228348     1.010663    -0.022100
  C     -2.777136     3.306104     0.399285
  C     -2.293618     4.551113     0.613811
  H      0.970288     1.161042    -0.019544
  H      8.354472     0.426075    -1.092737
  H      3.426279    -1.983694     0.072741
  H      2.585966    -4.657387     2.635314
  H      2.844362    -5.919873    -0.142153
  H      1.645451    -6.282154     1.097568
  H     -4.985429    -0.830076    -1.841138
  H     -7.833136    -0.350736    -1.565349
  H     -7.668377     0.280980     0.636793
```

```
  H     -8.147411    -1.447650     1.940249
  H     -5.206414    -1.457905     1.003226
  H     -3.213782     0.768441    -0.051304
  H     -3.836917     3.110805     0.385847
  H     -2.939122     5.400834     0.780031
  C      0.076700    -4.914241    -0.415038
  H     -0.551891    -4.054314    -0.618246
  H      0.228409    -5.472158    -1.342608
  H     -0.416979    -5.561772     0.307481
  C      6.458914    -1.644538    -1.150304
  H      6.127469    -2.220266    -0.288700
  H      5.864631    -1.922448    -2.019520
  H      7.505329    -1.859323    -1.349258
  C     -0.446201     6.154099     0.864857
  H      0.183017     6.167661     1.752348
  H      0.143917     6.482444     0.011852
  H     -1.288970     6.824197     1.005337
  C     -5.816487     1.096907    -2.275428
  H     -4.856373     1.599238    -2.395884
  H     -6.166843     0.790929    -3.262121
  H     -6.532631     1.805425    -1.856419
  C     -6.146679    -3.142640    -1.059272
  H     -6.313653    -4.045179    -1.644286
  H     -5.109867    -2.827657    -1.197927
  H     -6.316228    -3.361783    -0.005015
  H      1.480943    -0.486837    -0.351999
  H     -1.543046     0.288137    -0.167142
  H      2.500560     2.826916     0.099145
  H      3.943085    -5.597740     2.019154
  C     -5.542093     0.245964     2.266965
  H     -6.009972    -0.334165     3.064175
  H     -4.489895     0.377442     2.519554
  H     -6.020128     1.225932     2.223275


69
1VS8_gas_quartet E =  -2052.86495207 A.U.
  N      0.304617    -0.427001    -1.216572
  C     -0.775596    -1.265317    -1.192268
  N     -1.997899    -0.690330    -1.214297
  N     -0.674384    -2.580767    -1.166061
  C      0.604996    -3.003133    -1.173515
  C      1.772566    -2.248845    -1.195289
  C      1.660509    -0.821249    -1.219277
  O      2.540106     0.029863    -1.240478
  N      2.882252    -3.072325    -1.195848
  C      2.394365    -4.281312    -1.175259
  N      1.014414    -4.311277    -1.161573
  N      7.402859    -1.367812    -0.045148
  C      6.269869    -0.597353     0.021198
  N      5.203630    -1.053529    -0.653352
  N      6.187247     0.535995     0.698139
  C      7.348685     0.839485     1.315658
```

C	8.534305	0.136874	1.314892
C	8.609124	-1.076322	0.582030
O	9.570087	-1.843502	0.452401
N	9.494641	0.788502	2.076526
C	8.886694	1.856899	2.516655
N	7.584951	1.951313	2.089360
N	-9.229357	-1.596180	1.838602
C	-8.635023	-0.418152	2.248243
O	-8.962797	0.169086	3.265214
N	-7.640351	0.048032	1.423536
C	-7.190872	-0.532856	0.255768
O	-6.270020	-0.002581	-0.366875
C	-7.863002	-1.743305	-0.104891
C	-8.835864	-2.222014	0.678869
N	-1.499104	4.367838	-1.397605
C	-1.634751	2.969404	-1.233902
O	-0.615346	2.287124	-1.295589
N	-2.872607	2.456738	-1.021449
C	-3.932249	3.250649	-0.969935
N	-5.136348	2.699133	-0.744575
C	-3.825868	4.668298	-1.137502
C	-2.586849	5.169804	-1.347344
H	-2.788276	-1.299527	-1.113992
H	2.971550	-5.192037	-1.170946
H	4.409265	-0.462146	-0.792120
H	9.354914	2.584617	3.146020
H	-7.588507	-2.263957	-0.998465
H	-9.327476	-3.128045	0.391939
H	-5.959783	3.271137	-0.761669
H	-4.687403	5.315433	-1.095813
H	-2.407624	6.227033	-1.482891
C	-10.286953	-2.151019	2.695654
H	-9.888723	-2.353063	3.668020
H	-11.086804	-1.445009	2.777452
H	-10.655151	-3.058851	2.265336
C	-0.154816	4.893677	-1.625711
H	0.497093	4.623918	-0.797838
H	0.265446	4.467384	-2.534196
H	-0.212943	5.975500	-1.716110
C	0.153905	-5.476065	-1.131776
H	-0.408265	-5.517795	-0.198980
H	-0.552806	-5.444934	-1.959700
H	0.767915	-6.369439	-1.218704
C	6.627927	3.026527	2.387535
H	6.314486	3.488330	1.474630
H	5.776941	2.617178	2.890685
H	7.095738	3.756721	3.014332
H	-2.136644	0.306418	-1.116793
H	0.128459	0.578313	-1.238695
H	5.016449	-2.003433	-0.903676
H	-5.261212	1.698893	-0.650612
H	7.213053	-2.194377	0.484718
H	-6.964480	0.615442	1.893906

69
1VS8_cosmo_quartet E = -2052.97515823 A.U.

N	0.204030	-0.638510	-0.635847
C	-0.910069	-1.438801	-0.674609
N	-2.104814	-0.823246	-0.612687
N	-0.851027	-2.758170	-0.748961
C	0.408825	-3.227115	-0.779458
C	1.600541	-2.508058	-0.744414
C	1.530917	-1.087956	-0.665812
O	2.458745	-0.269752	-0.624551
N	2.686001	-3.364339	-0.799478
C	2.156401	-4.556747	-0.864665
N	0.777829	-4.542969	-0.856460
N	7.368618	-1.493418	0.182197
C	6.244732	-0.728839	-0.002137
N	5.185658	-1.353269	-0.539148
N	6.164061	0.552504	0.316015
C	7.317389	1.013631	0.844758
C	8.493526	0.332594	1.073382
C	8.566426	-1.041301	0.724643
O	9.519565	-1.819866	0.843673
N	9.447612	1.170066	1.634899
C	8.845412	2.324356	1.733647
N	7.553387	2.300144	1.268761
N	-9.298031	-1.050240	1.684793
C	-8.696258	0.191744	1.749119
O	-9.035641	1.050523	2.545259
N	-7.680047	0.392247	0.846925
C	-7.216046	-0.504952	-0.092689
O	-6.276636	-0.184099	-0.821669
C	-7.896939	-1.763249	-0.102469
C	-8.890730	-1.987742	0.764647
N	-1.181484	4.148213	-1.081199
C	-1.323301	2.775727	-0.829331
O	-0.302529	2.131231	-0.544305
N	-2.553226	2.215797	-0.905598
C	-3.617518	2.960506	-1.212049
N	-4.820385	2.378838	-1.272755
C	-3.499924	4.359294	-1.477901
C	-2.264884	4.901661	-1.398951
H	-2.923046	-1.400095	-0.727392
H	2.702296	-5.486923	-0.920813
H	4.282801	-0.881575	-0.561600
H	9.311034	3.199817	2.135739
H	-7.612305	-2.522196	-0.800954
H	-9.388596	-2.934580	0.741325
H	-5.633398	2.933199	-1.491997
H	-4.360064	4.961809	-1.728114
H	-2.081068	5.952275	-1.581734
C	-10.378921	-1.325790	2.642198
H	-10.001964	-1.240476	3.639965

H	-11.170893	-0.620749	2.498636
H	-10.750697	-2.316724	2.484901
C	0.155813	4.732818	-0.995990
H	0.560702	4.600839	0.005290
H	0.823801	4.245944	-1.703404
H	0.086101	5.792272	-1.226478
C	-0.114283	-5.687633	-0.917505
H	-0.744501	-5.725898	-0.030258
H	-0.751310	-5.626629	-1.798631
H	0.484198	-6.593416	-0.970743
C	6.605004	3.422303	1.221998
H	6.315068	3.602243	0.207862
H	5.739302	3.182113	1.803190
H	7.070292	4.299397	1.620907
H	-2.212488	0.186661	-0.685858
H	0.060407	0.379859	-0.584064
H	5.152271	-2.358329	-0.611177
H	-4.976393	1.403988	-1.049696
H	7.350010	-2.471626	-0.094526
H	-7.223542	1.300271	0.893696

69
3OAS_gas_quartet E = -2052.91098315 A.U.

N	0.671828	2.208473	0.712599
C	0.159877	3.422369	0.344794
N	-1.167463	3.583975	0.528355
N	0.888527	4.389746	-0.175359
C	2.184687	4.046151	-0.302725
C	2.808793	2.847782	0.031400
C	2.011961	1.799368	0.593069
O	2.337218	0.673583	0.959293
N	4.159172	2.912909	-0.254573
C	4.337164	4.108092	-0.746261
N	3.176183	4.848468	-0.803843
N	7.284178	-0.137250	-0.608365
C	6.110228	-0.720340	-0.205623
N	5.164961	0.093052	0.274432
N	5.870530	-2.015194	-0.267196
C	6.920981	-2.691205	-0.773041
C	8.135862	-2.207619	-1.206697
C	8.377737	-0.815642	-1.131839
O	9.389705	-0.190381	-1.464047
N	8.943740	-3.234088	-1.656427
C	8.218336	-4.299260	-1.496709
N	6.976067	-4.043270	-0.981681
N	-10.472010	-0.419279	-2.144844
C	-9.598237	-1.484400	-2.295283
O	-9.681360	-2.272551	-3.220719
N	-8.601902	-1.581654	-1.346621
C	-8.402128	-0.757118	-0.262231
O	-7.463472	-0.967177	0.504502
C	-9.359334	0.296903	-0.153043

```
  C    -10.342890     0.421419    -1.058041
  N     -1.282183    -0.967781     2.978465
  C     -2.081851    -0.038431     2.275285
  O     -1.554984     1.030610     1.936069
  N     -3.365290    -0.350093     2.013029
  C     -3.869865    -1.513783     2.398285
  N     -5.154751    -1.770532     2.108114
  C     -3.091891    -2.481448     3.107556
  C     -1.806012    -2.152733     3.369646
  H     -1.554422     4.492006     0.354290
  H      5.276228     4.514033    -1.086087
  H      5.160006     1.091345     0.100531
  H      8.559027    -5.282963    -1.744012
  H     -9.292035     0.993072     0.656724
  H    -11.058213     1.206998    -0.931223
  H     -5.708685    -1.132113     1.556492
  H     -3.498952    -3.429226     3.422655
  H     -1.137282    -2.815979     3.899244
  C      5.944979    -5.040613    -0.660673
  H      5.743839    -5.020176     0.390055
  H      5.049042    -4.813590    -1.199804
  H      6.291525    -6.014042    -0.938607
  C      3.013985     6.205062    -1.289383
  H      2.275894     6.234547    -2.089322
  H      2.681568     6.862446    -0.486884
  H      3.969346     6.558314    -1.669745
  C      0.108411    -0.615989     3.273556
  H      0.690473    -0.499953     2.360923
  H      0.141997     0.323254     3.821756
  H      0.543823    -1.405546     3.880899
  C    -11.591675    -0.302065    -3.090098
  H    -11.209791    -0.197479    -4.084145
  H    -12.200521    -1.180067    -3.032457
  H    -12.179715     0.556673    -2.841738
  H     -7.955505    -2.346029    -1.479902
  H     -5.562517    -2.657695     2.335041
  H      4.256506    -0.288817     0.484133
  H      7.404020     0.859720    -0.513671
  H      0.025807     1.543862     1.142709
  H     -1.704137     2.873351     1.007091


69
3OAS_cosmo_quartet E =  -2052.97626118 A.U.
  N      0.836022     1.848759     1.027304
  C      0.106695     3.007600     0.954249
  N     -1.142604     2.966393     1.461447
  N      0.582264     4.131904     0.448096
  C      1.850065     4.017311     0.014277
  C      2.671263     2.893088     0.027313
  C      2.151257     1.683221     0.575500
  O      2.701383     0.581346     0.690699
  N      3.906269     3.192853    -0.522018
```

C	3.821738	4.454529	-0.850656
N	2.599494	5.013757	-0.550993
N	7.457254	-0.000421	-1.259233
C	6.302242	-0.410224	-0.644139
N	5.362977	0.518427	-0.441779
N	6.074835	-1.647854	-0.251090
C	7.116975	-2.456285	-0.528034
C	8.313058	-2.150544	-1.139396
C	8.541977	-0.817131	-1.553295
O	9.536745	-0.343527	-2.111447
N	9.118209	-3.269008	-1.235982
C	8.409473	-4.215011	-0.698318
N	7.180439	-3.798036	-0.262857
N	-10.332801	-0.790633	-2.041510
C	-9.452434	-1.842908	-1.845906
O	-9.554614	-2.900347	-2.442488
N	-8.427213	-1.609397	-0.953439
C	-8.204068	-0.463390	-0.223775
O	-7.240753	-0.398144	0.538358
C	-9.168959	0.564379	-0.451642
C	-10.180399	0.371440	-1.312849
N	-1.968005	-1.938389	2.504130
C	-1.568140	-0.632125	2.189470
O	-0.375491	-0.330065	2.354049
N	-2.489317	0.240735	1.723151
C	-3.756869	-0.138026	1.544008
N	-4.632113	0.756960	1.076407
C	-4.189856	-1.465482	1.846238
C	-3.259176	-2.320945	2.324211
H	-1.715585	3.778744	1.290633
H	4.604074	5.039360	-1.311240
H	5.457762	1.481626	-0.728196
H	8.753257	-5.223938	-0.604615
H	-9.085054	1.495771	0.068311
H	-10.900072	1.152986	-1.439976
H	-4.313672	1.683926	0.838353
H	-5.213819	-1.769330	1.698586
H	-3.493846	-3.345016	2.583494
C	6.170254	-4.625051	0.412752
H	6.000371	-4.245965	1.398825
H	5.256212	-4.596363	-0.142774
H	6.518864	-5.634820	0.473988
C	2.175946	6.382800	-0.789394
H	1.339977	6.407365	-1.487019
H	1.868781	6.851155	0.144188
H	3.010469	6.937337	-1.210690
C	-0.960281	-2.867562	3.012358
H	-0.164042	-2.995203	2.281762
H	-0.524057	-2.482449	3.931702
H	-1.437258	-3.824378	3.205944
C	-11.481394	-1.004192	-2.933709
H	-11.130521	-1.246417	-3.915095
H	-12.078709	-1.809315	-2.559675

H	-12.071019	-0.112296	-2.975782
H	-7.775942	-2.381654	-0.830091
H	-5.593401	0.495515	0.890727
H	4.478465	0.286645	0.000028
H	7.550222	0.975084	-1.529793
H	0.402581	1.030263	1.482384
H	-1.618372	2.071961	1.585652

69
2AW4_gas_quartet E = -2052.79762580 A.U.

N	0.891718	2.188395	1.040276
C	0.388078	3.455903	0.956897
N	-0.752786	3.699824	1.650610
N	0.958392	4.426263	0.271806
C	2.095607	4.031767	-0.334865
C	2.679834	2.771495	-0.348515
C	2.048099	1.714802	0.385032
O	2.376746	0.541181	0.498297
N	3.834627	2.783071	-1.105037
C	3.941118	4.011914	-1.529111
N	2.913554	4.824355	-1.097571
N	6.958444	-0.583163	-1.649959
C	5.813865	-0.953591	-0.988394
N	4.774551	-0.112144	-1.086010
N	5.697104	-2.065380	-0.282770
C	6.835721	-2.791609	-0.293688
C	8.029439	-2.512054	-0.924997
C	8.142099	-1.312886	-1.675456
O	9.117510	-0.883316	-2.302934
N	8.956827	-3.517459	-0.691452
C	8.323977	-4.372850	0.065168
N	7.034252	-3.989412	0.348998
N	-9.839093	-1.618243	-2.248541
C	-9.286717	-2.379967	-1.237695
O	-9.600066	-3.540298	-1.036747
N	-8.352850	-1.729479	-0.471241
C	-7.925560	-0.424459	-0.611858
O	-7.051593	0.009882	0.139666
C	-8.551580	0.296463	-1.675526
C	-9.462843	-0.309925	-2.438785
N	-2.168962	-1.003365	3.015994
C	-1.741048	0.285871	2.636027
O	-0.587971	0.617184	2.909706
N	-2.616118	1.093743	1.984774
C	-3.835933	0.666077	1.687679
N	-4.659029	1.501293	1.030310
C	-4.296836	-0.637112	2.052830
C	-3.420778	-1.426007	2.716000
H	-1.362056	2.926738	1.893287
H	4.733875	4.395066	-2.151118
H	3.971859	-0.239287	-0.503303
H	8.763535	-5.278792	0.427072

```
  H      -8.290712     1.317837    -1.858910
  H      -9.919431     0.245101    -3.231498
  H      -5.553425     1.181493     0.688934
  H      -5.291045    -0.970962     1.804902
  H      -3.673726    -2.427496     3.033905
  C       6.056912    -4.703246     1.183314
  H       5.771496    -4.083566     2.007601
  H       5.192658    -4.938573     0.597995
  H       6.495148    -5.607225     1.551674
  C      -1.203574    -1.857771     3.703979
  H      -0.319473    -1.994013     3.084858
  H      -0.891550    -1.394094     4.637517
  H      -1.669129    -2.819201     3.905988
  C       2.723625     6.234324    -1.373294
  H       1.777793     6.398867    -1.888042
  H       2.717357     6.806908    -0.446716
  H       3.539073     6.582017    -2.003011
  C     -10.839745    -2.251817    -3.119249
  H     -10.402208    -3.096029    -3.609941
  H     -11.672616    -2.574023    -2.529825
  H     -11.172799    -1.546588    -3.851808
  H       4.846315     0.869695    -1.261664
  H      -4.290171     2.382520     0.720687
  H      -1.174665     4.593985     1.473915
  H       0.421487     1.525977     1.666198
  H      -7.816285    -2.181801    -1.183627
  H       6.734716    -0.531628    -2.623234


69
2AW4_cosmo_quartet E =  -2052.97883623 A.U.
  N       0.758967     2.067917     1.116250
  C       0.137639     3.278055     0.946752
  N      -1.069245     3.418489     1.537371
  N       0.674059     4.280667     0.274894
  C       1.885589     3.984498    -0.229540
  C       2.589293     2.786810    -0.143497
  C       2.004224     1.707122     0.584794
  O       2.449136     0.572326     0.786297
  N       3.795636     2.890428    -0.814394
  C       3.809216     4.110180    -1.283577
  N       2.678678     4.826057    -0.963062
  N       7.023150    -0.267592    -1.443308
  C       5.877775    -0.760003    -0.868376
  N       4.829592     0.075053    -0.826666
  N       5.768317    -1.979703    -0.370353
  C       6.915303    -2.681266    -0.497650
  C       8.110624    -2.283119    -1.058318
  C       8.215290    -0.969586    -1.585153
  O       9.190597    -0.426623    -2.117800
  N       9.047707    -3.304615    -0.996115
  C       8.418855    -4.286033    -0.407852
  N       7.112888    -3.975006    -0.074946
```

N	-9.756121	-1.349016	-2.378326
C	-9.202861	-2.271141	-1.511783
O	-9.504412	-3.451765	-1.521037
N	-8.282680	-1.756202	-0.633884
C	-7.869325	-0.442711	-0.538767
O	-7.006448	-0.138556	0.286001
C	-8.495343	0.447824	-1.465131
C	-9.393512	-0.023939	-2.331962
N	-2.150742	-1.273006	3.134312
C	-1.672244	-0.044998	2.658499
O	-0.492871	0.259763	2.899060
N	-2.505127	0.755977	1.956752
C	-3.752557	0.368329	1.682866
N	-4.533505	1.186309	0.970641
C	-4.262135	-0.885507	2.140583
C	-3.424435	-1.661599	2.863460
H	4.602899	4.551813	-1.867593
H	4.032046	-0.151627	-0.245133
H	8.832923	-5.257876	-0.183048
H	-8.224780	1.491896	-1.450248
H	-9.887100	0.613065	-3.054207
H	-5.460209	0.894332	0.681219
H	-5.270503	-1.194773	1.917828
H	-3.722684	-2.624848	3.256130
C	6.147053	-4.830580	0.612637
H	5.905921	-4.422232	1.593085
H	5.233090	-4.909007	0.027039
H	6.584426	-5.818365	0.731570
C	-1.239905	-2.121881	3.900448
H	-0.372276	-2.382572	3.297645
H	-0.895447	-1.596732	4.788908
H	-1.769425	-3.024949	4.191228
C	2.376544	6.200461	-1.324319
H	1.450473	6.246572	-1.894987
H	2.270335	6.814108	-0.430986
H	3.191011	6.588291	-1.930586
C	-10.742516	-1.829540	-3.356600
H	-10.295638	-2.588464	-3.995119
H	-11.598522	-2.262791	-2.843597
H	-11.064190	-0.985885	-3.959075
H	4.909905	1.065582	-1.027475
H	-4.149790	2.049687	0.617392
H	0.302171	1.369526	1.723248
H	-7.859384	-2.425975	0.004752
H	7.025364	0.685280	-1.797540
H	-1.615302	2.581580	1.744262
H	-1.579763	4.254637	1.295867

38
1EVV_carbonyl_gas E = -1092.95948598 A.U.

C	-5.526309	-1.849137	0.108459
N	-4.763884	-0.644237	-0.156075

C	-5.224634	0.558239	-0.667362
N	-4.291955	1.459758	-0.786678
C	-3.147882	0.837571	-0.333828
C	-1.804984	1.322345	-0.211249
O	-1.347302	2.421455	-0.489695
N	-0.960442	0.311118	0.326141
C	-1.343608	-0.959753	0.664047
N	-0.378665	-1.771664	1.176790
N	-2.575432	-1.403140	0.545241
C	-3.423338	-0.469290	0.061808
C	4.193968	2.134131	0.394102
N	4.001588	0.709501	0.088356
C	2.762465	0.117068	0.424682
O	1.901097	0.805634	0.971307
N	2.575319	-1.197983	0.129193
C	3.521175	-1.897915	-0.475131
N	3.264975	-3.199969	-0.738753
C	4.779674	-1.329392	-0.830465
C	4.961476	-0.019899	-0.523919
O	1.284768	3.534825	0.024663
H	-0.662325	-2.726587	1.317130
H	0.597913	-1.594381	0.962399
H	0.006009	0.583090	0.514717
H	-6.263883	0.690250	-0.927895
H	-5.132585	-2.686074	-0.469347
H	-5.483245	-2.105685	1.167580
H	-6.563000	-1.675456	-0.175188
H	3.896333	-3.753626	-1.289389
H	2.337556	-3.552003	-0.570536
H	5.879763	0.502086	-0.756953
H	5.554320	-1.900341	-1.319543
H	3.479463	2.741849	-0.160874
H	4.038979	2.301944	1.458678
H	5.211770	2.408541	0.124840
H	0.377510	3.385618	-0.290059
H	1.428357	2.777983	0.607861

38

1EVV_carbonyl_cosmo E = -1093.00373618 A.U.

C	-5.490991	-2.001671	0.296083
N	-4.786662	-0.757807	0.020604
C	-5.338461	0.451404	-0.349248
N	-4.449729	1.393384	-0.531094
C	-3.239389	0.778579	-0.269502
C	-1.910792	1.289032	-0.292715
O	-1.540706	2.439312	-0.564556
N	-0.972477	0.300734	0.049488
C	-1.271306	-1.003497	0.368614
N	-0.232439	-1.799416	0.703861
N	-2.503452	-1.478138	0.391566
C	-3.433803	-0.557863	0.075253
C	4.263857	2.208796	0.325452

N	4.085166	0.770727	0.103443
C	2.787200	0.246702	0.103107
O	1.829108	1.020003	0.296169
N	2.612461	-1.077370	-0.102851
C	3.659630	-1.876569	-0.314727
N	3.425290	-3.180383	-0.523756
C	4.994078	-1.374203	-0.320978
C	5.150593	-0.046607	-0.106963
O	0.830237	3.696497	0.325102
H	-0.447138	-2.778023	0.809609
H	0.723610	-1.559666	0.432248
H	0.014611	0.588666	0.089311
H	-6.405490	0.562719	-0.464470
H	-5.178338	-2.776739	-0.402953
H	-5.287279	-2.333365	1.313564
H	-6.558400	-1.828086	0.183482
H	4.177149	-3.826746	-0.692280
H	2.481077	-3.529506	-0.536918
H	6.122398	0.425593	-0.093761
H	5.844819	-2.016125	-0.489750
H	3.754385	2.773823	-0.453250
H	3.857234	2.490059	1.295386
H	5.326447	2.433420	0.298987
H	-0.017388	3.346219	-0.007696
H	1.372374	2.894538	0.399680

38
1YIJ_amino_gas E = -1092.95995000 A.U.

C	4.035299	-2.871101	0.538071
N	4.006304	-1.429567	0.289409
C	2.745215	-0.785482	0.316241
O	1.746814	-1.462703	0.554686
N	2.698358	0.549387	0.080629
C	3.796687	1.236353	-0.209926
N	3.667511	2.545914	-0.478148
C	5.086620	0.611424	-0.230828
C	5.129773	-0.718564	0.023670
C	-5.456129	1.567161	0.810134
N	-0.990903	-0.628138	-0.101049
C	-1.275482	0.586599	0.446158
N	-0.202216	1.394831	0.782482
N	-2.487922	1.050327	0.620196
C	-3.441790	0.192412	0.187579
C	-3.283352	-1.049987	-0.416889
C	-1.952829	-1.567175	-0.601418
O	-1.580258	-2.619627	-1.078609
N	-4.509236	-1.598198	-0.725100
C	-5.377011	-0.713498	-0.319170
N	-4.793649	0.406493	0.246290
O	0.952969	3.519090	-0.841839
H	3.620932	-3.088711	1.521341
H	4.470403	3.086266	-0.747634

```
H       5.987616     1.164651    -0.449901
H       6.057434    -1.275734     0.021526
H      -5.324804     1.602994     1.892834
H      -0.399307     2.000283     1.566725
H      -6.449884    -0.802697    -0.398750
H       0.412065     2.813567    -0.429585
H       0.540785     3.705997    -1.691112
H      -6.519030     1.509346     0.581354
H      -5.045711     2.480105     0.379060
H       5.066787    -3.214167     0.486268
H       3.432748    -3.390700    -0.205626
H      -0.028121    -0.976084    -0.049852
H       0.726266     0.973548     0.783912
H       2.735886     2.951045    -0.599169
```

```
38
1YIJ_amino_cosmo E =  -1092.99908611 A.U.
C      -3.983278    -2.927819    -0.491800
N      -3.942867    -1.473709    -0.318239
C      -2.699262    -0.860838    -0.103012
O      -1.682748    -1.574424    -0.064302
N      -2.649302     0.482089     0.052398
C      -3.764836     1.217117     0.039829
N      -3.650184     2.537439     0.238804
C      -5.042937     0.620253    -0.186477
C      -5.075064    -0.722618    -0.358004
C       5.419078     1.564728    -0.963404
N       0.974530    -0.573592     0.238036
C       1.233167     0.652667    -0.313482
N       0.156394     1.461763    -0.552924
N       2.444603     1.094657    -0.574884
C       3.412827     0.219666    -0.231906
C       3.268139    -1.032085     0.359913
C       1.950914    -1.517299     0.626517
O       1.610134    -2.596406     1.111076
N       4.503166    -1.616787     0.563504
C       5.359109    -0.737658     0.107631
N       4.760589     0.399760    -0.389903
O      -1.033956     3.587395     1.156671
H      -3.380111    -3.218661    -1.350190
H      -4.473563     3.114108     0.282069
H      -5.949644     1.205222    -0.210451
H      -5.997215    -1.260129    -0.528834
H       5.084655     1.720891    -1.988343
H       0.297708     2.140246    -1.287451
H       6.432310    -0.849092     0.101343
H      -0.432714     2.909706     0.797250
H      -0.906714     3.564754     2.112386
H       6.492574     1.392701    -0.960016
H       5.196528     2.453719    -0.374086
H      -5.014866    -3.229386    -0.652000
H      -3.591983    -3.421726     0.396055
```

```
  H     -0.004138    -0.901778     0.269129
  H     -0.787204     1.070632    -0.477772
  H     -2.758715     2.938265     0.530980


41
1EHZ_gas E =  -1169.43796937 A.U.
  C     -5.599384     1.603282     0.681723
  N     -4.859045     0.459887     0.181141
  C     -5.365744    -0.706438    -0.365431
  N     -4.442727    -1.560983    -0.707479
  C     -3.257691    -0.942895    -0.374450
  C     -1.901705    -1.399696    -0.482594
  O     -1.468753    -2.460517    -0.901018
  N     -1.004985    -0.404667     0.011100
  C     -1.364590     0.815411     0.504661
  N     -0.338836     1.677796     0.848206
  N     -2.602292     1.226052     0.617092
  C     -3.498277     0.311091     0.179984
  H     -0.582048     2.300947     1.605092
  H      0.606579     1.297242     0.883948
  H     -0.027942    -0.696441     0.112172
  H     -6.429220    -0.850785    -0.481310
  H     -5.207111     2.522472     0.247562
  H     -6.645497     1.492588     0.401467
  H     -5.525069     1.670027     1.768270
  C      4.056615    -2.511503     0.632772
  N      3.960519    -1.074764     0.345047
  C      2.697839    -0.455119     0.438201
  O      1.725094    -1.133699     0.790628
  N      2.593372     0.861788     0.145148
  C      3.650277     1.561562    -0.257542
  N      3.467221     2.851753    -0.579358
  C      4.949190     0.966166    -0.339838
  C      5.042501    -0.350229    -0.033014
  H      3.665688    -2.713198     1.628590
  H      3.476621    -3.084293    -0.090343
  H      5.103996    -2.802205     0.584765
  H      5.818446     1.527892    -0.647602
  H      5.980118    -0.887622    -0.080726
  H      4.235238     3.392794    -0.935737
  H      2.520426     3.229277    -0.665039
  O      1.152576    -3.732099    -0.406305
  H      0.209377     3.072078    -0.426008
  H      0.291614     3.924309    -1.719697
  O      0.728446     3.769692    -0.876231
  H      0.266159    -3.570276    -0.763983
  H      1.276937    -2.979651     0.188875

41
1EHZ_2HOH_cosmo E =  -1169.47941069 A.U.
  C     -5.480109    -1.757639    -0.943608
  N     -4.814481    -0.579702    -0.405070
```

C	-5.406294	0.575701	0.059410
N	-4.546405	1.463852	0.488012
C	-3.314868	0.866539	0.300850
C	-1.998341	1.350936	0.546223
O	-1.663110	2.451769	0.999323
N	-1.024535	0.398468	0.189416
C	-1.288695	-0.847345	-0.321468
N	-0.218247	-1.668794	-0.529344
N	-2.502432	-1.289281	-0.569697
C	-3.466664	-0.402366	-0.253573
H	-0.363419	-2.382092	-1.229175
H	0.731193	-1.291750	-0.457652
H	-0.043583	0.709661	0.222902
H	-6.478945	0.692096	0.051060
H	-5.147595	-1.944902	-1.963867
H	-5.261247	-2.629819	-0.328493
H	-6.552547	-1.579524	-0.943955
C	4.073259	2.576587	-0.639825
N	3.972310	1.132636	-0.409589
C	2.712812	0.575052	-0.164821
O	1.714720	1.324139	-0.155186
N	2.609535	-0.753051	0.047620
C	3.696812	-1.531813	0.064789
N	3.532781	-2.835078	0.322172
C	4.994867	-0.993490	-0.190060
C	5.076670	0.337987	-0.418808
H	3.430208	2.866258	-1.468620
H	3.772342	3.122618	0.253314
H	5.105065	2.817702	-0.879793
H	5.878820	-1.612707	-0.191220
H	6.016873	0.833769	-0.614124
H	4.334135	-3.440326	0.386498
H	2.628477	-3.189879	0.634985
O	0.691638	3.915767	0.404129
H	0.314249	-3.076186	0.905704
H	0.777971	-3.666630	2.253963
O	0.897135	-3.749335	1.300446
H	-0.151614	3.500199	0.661006
H	1.230167	3.148552	0.148479

48

1FFK_gas E = -1738.98098413 A.U.

O	3.296120	-2.907371	1.295305
P	3.208820	-2.377722	-0.264424
O	4.608587	-1.514167	-0.274082
O	2.116547	-1.339801	-0.371027
O	3.278372	-3.543056	-1.193903
N	0.959383	4.677260	0.125980
C	0.292075	3.429311	-0.024975
O	-0.935590	3.442666	-0.082826
N	1.043808	2.312035	-0.095328
C	2.373528	2.348881	-0.025119

N	3.046787	1.207871	-0.101306
C	3.066355	3.602781	0.129263
C	2.313341	4.721696	0.198261
N	-2.628874	0.875329	-0.107949
C	-1.905624	-0.286195	-0.268821
N	-0.585186	-0.166275	-0.438426
N	-2.474670	-1.483559	-0.273529
C	-3.805339	-1.433779	-0.112371
C	-4.634597	-0.329410	0.057418
C	-4.034436	0.976105	0.069434
O	-4.558493	2.069471	0.204256
N	-5.952145	-0.725552	0.189625
C	-5.916237	-2.025321	0.102040
N	-4.639935	-2.525659	-0.082593
H	2.591414	0.274911	-0.203473
H	4.142385	3.651917	0.188077
H	2.750816	5.704181	0.314736
H	0.008826	-0.984042	-0.475961
H	-6.769680	-2.682884	0.161743
C	-4.229213	-3.905492	-0.220005
H	-3.151297	-3.916723	-0.362314
H	-4.475087	-4.480366	0.674750
H	-4.706157	-4.372964	-1.083251
C	0.136511	5.875498	0.199564
H	-0.456218	5.984523	-0.707406
H	-0.555237	5.808529	1.037657
H	0.784390	6.741448	0.323103
C	5.838295	-2.226600	-0.312521
H	5.814439	-2.999598	-1.082082
H	6.625945	-1.506782	-0.539914
H	6.048246	-2.692563	0.654164
H	-0.105692	0.724425	-0.335061
H	4.049293	1.204536	-0.047842
H	-2.128785	1.765105	-0.110802
C	2.368100	-3.903112	1.701263
H	2.630515	-4.196288	2.718346
H	1.345782	-3.514357	1.698536
H	2.418726	-4.775446	1.045677

48
1FFK_cosmo E = -1739.08142271 A.U.

O	3.073936	-2.495099	1.271758
P	3.256675	-2.144447	-0.315577
O	4.690371	-1.362872	-0.349436
O	2.246500	-1.070251	-0.640815
O	3.312880	-3.411713	-1.118210
N	0.361343	4.790121	0.168303
C	-0.104074	3.481833	-0.040332
O	-1.328511	3.296288	-0.109889
N	0.798051	2.483459	-0.157210
C	2.109172	2.719581	-0.071876

N	2.950681	1.689891	-0.194354
C	2.606828	4.042598	0.144199
C	1.695009	5.034530	0.255338
N	-2.590193	0.671059	-0.155440
C	-1.768487	-0.409200	-0.382917
N	-0.483156	-0.136824	-0.646483
N	-2.205938	-1.660089	-0.371298
C	-3.524372	-1.754187	-0.120402
C	-4.438639	-0.732060	0.118459
C	-3.969614	0.616900	0.110675
O	-4.603811	1.655391	0.300077
N	-5.702892	-1.250842	0.334005
C	-5.547070	-2.543307	0.226886
N	-4.247993	-2.915091	-0.048368
H	2.627779	0.729211	-0.348212
H	3.664643	4.246534	0.214752
H	1.981266	6.065358	0.418363
H	0.202176	-0.881237	-0.670571
H	-6.325181	-3.284527	0.334335
C	-3.739202	-4.262924	-0.226271
H	-3.297432	-4.375957	-1.215275
H	-2.981515	-4.487353	0.523342
H	-4.563778	-4.963488	-0.121167
C	-0.621831	5.864072	0.292422
H	-1.228357	5.924241	-0.608985
H	-1.281225	5.674696	1.137170
H	-0.095013	6.802450	0.442449
C	5.898501	-2.061763	-0.042590
H	5.969571	-2.990131	-0.610952
H	6.725263	-1.408514	-0.313983
H	5.957991	-2.282892	1.025146
H	-0.099559	0.797941	-0.494711
H	3.943876	1.850121	-0.133622
H	-2.169413	1.608266	-0.163730
C	3.237712	-3.824538	1.774710
H	2.914555	-3.807790	2.813802
H	2.629934	-4.532759	1.213172
H	4.280456	-4.142402	1.730554

51
3CCL_gas E = -795.783361835 A.U.

C	3.050545	-2.625331	1.696740
O	3.870866	-2.755184	0.543692
P	3.980946	-1.502131	-0.510026
C	6.267869	-0.645839	0.494453
O	4.872411	-0.406413	0.364485
O	2.626008	-0.837736	-0.615026
O	4.760247	-1.978529	-1.681746
C	-1.571679	5.324510	0.252704
N	-0.413813	4.433954	0.232019
C	-0.557567	3.160125	-0.366729

O	-1.649636	2.867670	-0.866712
N	0.495290	2.322494	-0.359782
C	1.665595	2.664673	0.180793
N	2.652359	1.777582	0.171391
C	1.846836	3.968061	0.761355
C	0.780549	4.797692	0.763399
C	-2.514710	-5.052803	0.140727
N	-2.431203	-0.047378	-0.450906
C	-1.420667	-0.967724	-0.640839
N	-0.240419	-0.494095	-1.063205
N	-1.596487	-2.265091	-0.443466
C	-2.842129	-2.572306	-0.051871
C	-3.928493	-1.732251	0.179900
C	-3.747083	-0.325512	-0.017670
O	-4.556610	0.582347	0.140226
N	-5.032394	-2.461471	0.579336
C	-4.622532	-3.697508	0.586411
N	-3.299051	-3.839438	0.208769
O	-4.380223	3.471371	-0.088629
H	3.426072	-1.844696	2.365424
H	6.725724	-0.812106	-0.481470
H	6.461741	-1.518683	1.124757
H	-1.825764	5.642807	-0.758324
H	3.538745	1.997535	0.588527
H	2.787624	4.270844	1.193730
H	0.823798	5.789350	1.192185
H	-1.904206	-5.184096	1.036136
H	0.577488	-1.090773	-1.014619
H	-5.220549	-4.557315	0.845818
H	-4.588043	2.540852	0.098004
H	-3.540773	3.390347	-0.561002
H	-3.182152	-5.906719	0.036557
H	-1.849734	-5.005847	-0.719113
H	-2.436777	4.811913	0.668563
H	-1.328993	6.196561	0.856460
H	6.709302	0.234372	0.964868
H	2.021434	-2.388502	1.423869
H	3.077875	-3.581924	2.219255
H	-2.224524	0.935418	-0.633151
H	-0.035439	0.498727	-0.972596
H	2.549266	0.805068	-0.189141

51
3CCl_cosmo E = -1815.56217016 A.U.

C	4.189198	-0.809332	2.225935
O	4.078292	-1.906433	1.314301
P	3.787008	-1.620920	-0.267669
C	6.385726	-1.513079	-0.789815
O	5.129929	-0.827891	-0.753312
O	2.672786	-0.610528	-0.416211
O	3.680825	-2.968195	-0.915892
C	-1.855703	5.390971	0.076240

```
  N     -0.632460     4.589646     0.083888
  C     -0.724080     3.222177    -0.199242
  O     -1.843567     2.737029    -0.448844
  N      0.396839     2.475580    -0.191774
  C      1.588122     3.016798     0.075858
  N      2.662166     2.224420     0.072550
  C      1.712551     4.412658     0.358719
  C      0.578593     5.148006     0.351015
  C     -2.205731    -5.211083     0.049035
  N     -2.458185    -0.156028    -0.211037
  C     -1.364206    -0.981679    -0.363560
  N     -0.199100    -0.383905    -0.645662
  N     -1.442504    -2.299994    -0.266454
  C     -2.687079    -2.738425    -0.010775
  C     -3.850693    -1.992652     0.160532
  C     -3.768034    -0.574583     0.062189
  O     -4.679991     0.251249     0.189478
  N     -4.928664    -2.822406     0.410308
  C     -4.423072    -4.026182     0.388292
  N     -3.066685    -4.045217     0.137749
  O     -4.684216     3.046604    -0.360790
  H      5.035282    -0.169896     1.966974
  H      6.327789    -2.394361    -1.429191
  H      6.694528    -1.816337     0.212483
  H     -2.275864     5.431980    -0.927649
  H      3.567207     2.618365     0.278314
  H      2.668785     4.865001     0.574909
  H      0.577904     6.210113     0.557916
  H     -1.436935    -5.177817     0.819787
  H      0.664476    -0.911753    -0.595046
  H     -4.970829    -4.943916     0.543900
  H     -4.749268     2.103803    -0.130731
  H     -3.730651     3.161711    -0.482175
  H     -2.811822    -6.102828     0.187223
  H     -1.723365    -5.253571    -0.926303
  H     -2.594211     4.951568     0.742656
  H     -1.614313     6.396616     0.408835
  H      7.116826    -0.816391    -1.194282
  H      3.276712    -0.212351     2.228963
  H      4.349768    -1.232275     3.215288
  H     -2.304108     0.853207    -0.295387
  H     -0.082361     0.624513    -0.533000
  H      2.603481     1.217502    -0.111783
```

…….

Modeled geometries:

```
50
1N78_nowater E =  -1470.82602127 A.U.
  N     -0.541131    -2.278843     0.205544
  C      0.090778    -1.331253     0.955969
```

N	-0.693501	-0.462462	1.636845
N	1.408302	-1.247708	1.064967
C	2.051796	-2.261729	0.428757
C	1.509068	-3.270531	-0.358205
C	0.084062	-3.319524	-0.543649
O	-0.594342	-4.085111	-1.198030
N	2.498164	-4.113257	-0.816747
C	3.597530	-3.630265	-0.312574
N	3.398165	-2.502024	0.459724
N	3.344191	3.154971	-0.915778
C	3.167165	1.869710	-0.395439
O	3.965714	0.984726	-0.621924
N	2.036073	1.700558	0.378002
C	1.087602	2.671844	0.682643
O	0.114232	2.408783	1.385705
C	1.356730	3.970617	0.109737
C	2.452362	4.153020	-0.652186
N	-5.235385	-0.233295	-0.536111
C	-3.930139	-0.483020	-0.041798
O	-3.532085	-1.640764	0.000319
N	-3.191953	0.582536	0.362251
C	-3.659841	1.816537	0.295256
N	-2.858331	2.817798	0.703458
C	-4.976177	2.093669	-0.192405
C	-5.714220	1.030182	-0.592701
H	-1.610663	-0.247099	1.252833
H	4.586782	-4.036316	-0.452831
H	0.670366	4.775815	0.309592
H	2.685068	5.109680	-1.097941
H	-3.177505	3.767402	0.673153
H	-5.371154	3.095565	-0.247348
H	-6.718134	1.141697	-0.977833
H	-1.558449	-2.245675	0.145270
H	-0.214629	0.354152	1.984973
H	-1.908464	2.632033	1.005202
H	1.885523	0.737842	0.721047
C	4.399367	-1.766290	1.208183
H	4.577770	-0.790911	0.760730
H	4.068571	-1.634767	2.237891
H	5.323482	-2.340642	1.206196
C	-6.020344	-1.385510	-0.971124
H	-5.494028	-1.917828	-1.760572
H	-6.163624	-2.074934	-0.141577
H	-6.984084	-1.037721	-1.335073
C	4.528704	3.370620	-1.745036
H	5.433337	3.192859	-1.166764
H	4.524992	2.685272	-2.589717
H	4.517875	4.396124	-2.103425

50

GCU_1N78_nowater_cosmo E = -1470.86878659 A.U.

N	2.677442	0.957423	0.065439
C	1.331026	1.229506	0.107672

N	0.507131	0.176601	0.196934
N	0.842262	2.460539	0.077520
C	1.799550	3.403536	0.006626
C	3.180690	3.237378	-0.038532
C	3.708707	1.910756	-0.010447
O	4.884482	1.547062	-0.043529
N	3.815839	4.464260	-0.107902
C	2.842772	5.335668	-0.104309
N	1.592838	4.756745	-0.036769
N	-6.150887	0.003895	-0.012184
C	-5.196685	1.012607	0.000773
O	-5.490853	2.193901	-0.001968
N	-3.891203	0.566814	0.016777
C	-3.445826	-0.748703	0.021101
O	-2.235771	-0.984622	0.036906
C	-4.501591	-1.724496	0.006173
C	-5.787227	-1.313277	-0.009331
N	3.237314	-4.089115	-0.005300
C	2.663814	-2.808776	0.043541
O	3.420728	-1.829562	0.112518
N	1.316378	-2.698002	0.014430
C	0.540032	-3.779710	-0.059935
N	-0.786726	-3.611348	-0.085047
C	1.098610	-5.093498	-0.112082
C	2.446842	-5.190524	-0.081589
H	0.838471	-0.786105	0.135565
H	2.955382	6.408861	-0.148298
H	-4.252794	-2.773819	0.009068
H	-6.607893	-2.018865	-0.020166
H	-1.393994	-4.413996	-0.140134
H	0.476131	-5.973420	-0.172642
H	2.957738	-6.143864	-0.116218
H	2.970735	-0.027088	0.088295
H	-0.489640	0.322171	0.156023
H	-1.210802	-2.689839	-0.044500
H	-3.181975	1.297076	0.025854
C	0.309940	5.435472	-0.011608
H	-0.304953	5.118703	-0.852811
H	-0.221458	5.214609	0.913221
H	0.480771	6.506999	-0.077908
C	4.694728	-4.195443	0.027813
H	5.129239	-3.666593	-0.818044
H	5.082713	-3.754575	0.943817
H	4.967792	-5.246115	-0.017380
C	-7.556557	0.414940	-0.029423
H	-7.782923	1.011633	0.851628
H	-7.761842	1.010139	-0.916665
H	-8.175040	-0.477435	-0.036052

H2U to U modification
74

H2UtoU_gasphase E = -949.555230329 A.U.

N	-2.737259	2.375545	-0.179334
C	-2.783022	1.013121	-0.207806
N	-1.945760	0.390912	-1.071447
N	-3.611126	0.301033	0.541319
C	-4.374861	1.070943	1.359769
C	-4.384680	2.456869	1.482606
C	-3.502124	3.234189	0.659084
O	-3.335829	4.437806	0.603606
N	-5.284450	2.853902	2.447436
C	-5.796578	1.742448	2.891298
N	-5.287370	0.616645	2.272847
N	-2.941136	-4.357909	-1.448141
C	-2.827656	-3.086456	-0.893814
O	-1.732301	-2.553700	-0.773235
N	-4.004008	-2.500007	-0.508190
C	-5.291943	-3.040181	-0.640890
O	-6.271713	-2.425363	-0.261241
C	-5.316411	-4.359471	-1.245660
C	-4.167199	-4.950828	-1.612209
C	5.462540	-0.365667	1.689008
O	5.240803	-0.447640	0.283692
C	6.631076	-1.285708	1.970737
O	5.995908	-2.515438	2.294691
C	7.356263	-1.359958	0.613719
O	8.110265	-2.587713	0.512417
C	6.194087	-1.340685	-0.327085
N	1.414003	4.178994	-1.686195
C	0.487481	3.109855	-1.729054
O	-0.681631	3.366943	-2.001835
N	0.936744	1.858834	-1.456890
C	2.207425	1.642582	-1.160269
N	2.583379	0.376834	-0.894202
C	3.170344	2.699042	-1.127385
C	2.713552	3.946152	-1.394795
H	-1.156184	0.910411	-1.430443
H	-6.548033	1.656916	3.660213
H	-3.922705	-1.552185	-0.096788
H	-6.269861	-4.841359	-1.382464
H	-4.144399	-5.934209	-2.059695
H	4.575216	-0.571177	2.242764
H	7.279268	-0.981547	2.790897
H	8.004061	-0.488191	0.489141
H	8.920513	-2.405563	0.029959
H	5.793441	-2.357859	-0.379531
H	3.508120	0.149183	-0.555687
H	4.207774	2.518237	-0.898421
H	3.362068	4.810912	-1.385329
C	-1.706293	-5.012897	-1.870469
H	-1.214710	-4.434340	-2.650704
H	-1.020715	-5.103991	-1.030580
H	-1.951512	-6.001008	-2.249780
C	-5.628877	-0.763128	2.561911

H	-6.032742	-1.255364	1.679392
H	-4.748888	-1.308564	2.905572
H	-6.376174	-0.773441	3.352491
C	0.898759	5.523149	-1.940690
H	0.495987	5.585481	-2.949677
H	0.094298	5.748228	-1.243946
H	1.709008	6.237958	-1.819864
C	5.718450	1.086977	2.037623
H	4.882253	1.704003	1.705768
H	5.804506	1.206494	3.119770
H	6.627715	1.485187	1.581557
C	4.603740	-2.430478	1.979182
H	4.315742	-3.490952	1.937658
H	3.998727	-2.038664	2.796701
H	4.315923	-2.031488	1.022948
H	-1.802990	-0.601175	-0.925532
H	1.863642	-0.324821	-0.866692
H	-2.093003	2.844100	-0.816843
C	6.331872	-0.760295	-1.718271
H	7.005394	-1.357182	-2.337091
H	5.361017	-0.742777	-2.210893
H	6.716426	0.258650	-1.669285
